# Atomistic study on mechanical properties of Al matrix composite with different combining forms of reinforcements

**DOI:** 10.1371/journal.pone.0329889

**Published:** 2025-08-11

**Authors:** Yongchao Zhu, Na Li, Lijuan Sun, Baolin Wang, Yanlong Gao, Songtao Li

**Affiliations:** 1 Department of Railway Engineering, Zhengzhou Railway Vocational and Technical College, Zhengzhou, China; 2 School of Mechanics and Engineering Science, Zhengzhou University, Zhengzhou, China; 3 Henan Jiaoyuan Engineering Technology Group Co. Ltd, Zhengzhou, China; IIIT Kurnool: Indian Institute of Information Technology Design and Manufacturing Kurnool, INDIA

## Abstract

Models of matrix composite (MMC) are built through molecular dynamic (MD) simulation to study the effect of different combining forms of reinforcements. Diamond particle and graphene nanoplate (GNP) are selected as the two kinds of reinforcements, forming six combinations by changing the location and orientation of them. Then, the same sintering processes are conducted to achieve sintered composites. Bulk volume and Al volume of sintered composite reveal that a compacter structure can be produced in the model with two GNPs those are not parallel, or in the model where the diamond particle is out of the GNP plane. Structural analysis indicates that the ratio of arranged atoms rather than nanopore has a greater impact on Al volume. Tensile results show that the model reinforced by both GNP and diamond in a same plane gives the best performance in both strength and ductility, regardless of its low ratio of arranged atoms that may lead to a further improvement at the larger scale. In other words, GNP can play its role very well along the GNP plane, and diamond particle can improve the property vertical to GNP. This combined strengthening mechanism can be well presented by evolution of atomic configurations.

## 1. Introduction

In the investigations on metal matrix composite (MMC), many efforts have been made to study the effect of different reinforcements. However, their works are mainly conducted by addition of single reinforcing material, to improve the mechanical property. In general, the reinforcements in MMC can be classified into three kinds: three-dimensional (3D) particles, two-dimensional (2D) nanoplates, and one-dimensional (1D) nanotubes. With the development of research, almost every kind of reinforcement has been studied thoroughly.

3D ceramic particles are most commonly used to improve MMC, and the types of them are very rich. Sharma et al. investigated the properties of aluminium alloy composites reinforced with a rise in the percentage of Si_3_N_4_ [1]. Kumar et al. changed the amount of Si_3_N_4_ from 0 to 10 wt.% in Al matrix to assess the mechanical and tribological characteristics of composites [2]. Wang et al. evaluated the impact of different volume fractions of SiC on 2014Al matrix composites, finding that the 0.5 vol.% of SiC can result in the highest performance [3]. Zhang et al. incorporated ZrC into the AlCoCrFeNi high-entropy alloy using the spark plasma sintering, to enhance the wear resistance of the composite [4]. Jin et al. uniformly distributed micron- and nano-double-sized TiC particles in Al–Cu matrix composite using a wire and arc additive manufacturing process [5]. Hou et al. enabled a mechanical strong yet ductile CoCrNi/Cr2B composite by in-situ formed borides during laser powder bed fusion [6]. Generally, these early studies focus on obtaining the optimal amount of reinforced particles to produce MMC with high performance, while the recent researches concentrate on preparation of MMC with new reinforcement or by good dispersible methods.

In view of the specific performance, low-dimensional materials attract more attentions in promoting the property of MMC, and graphene or carbon nanotube (CNT) is commonly selected as the 2D or 1D reinforcement. Zhou et al. fabricated nickel based composites with well-preserved graphene actualizing the superior tensile strength and ductility [7]. Liu et al prepared the laminated graphene(Ni)/ high-entropy alloy matrix composites possessing excellent strength and good plasticity [8]. Gao et al. confirmed a clear improvement on the strength and plasticity in nickel-based superalloy FGH95 reinforced with the graphene (0.01 and 0.03 wt.%) [9]. Lou et al. prepared a 0.5 wt.% graphene-reinforced aluminum composite to study the effects of the extrusion ratio on composite [10]. Tian et al. discussed the effect of good interfacial bonding on the pull-out process by Ag-CNTs-reinforced copper matrix composites [11]; Kim achieved Al-CNT composite with high hardness and electrical conductivity by controlling Al_4_C_3_ formation [12]. Cao et al. applied a modified-ball-milling-involved powder metallurgy process to embed CNTs into matrix grains, improving the ductility of CNTs/Al composites [13]. These investigations mainly aim at proving the superiority of low-dimensional materials over traditional materials.

As reported above, single kind of reinforcement may have a limitation in promoting the mechanical property of MMC. Therefore, some researchers pay close attention to addition of multiple reinforcements in MMC. Mustu et al. introduced 0.5 wt.% GNPs into ZK60/(15 wt.%)TiB_2_, leading to a rise on wear performance rather than mechanical property [14]. Elkady et al. brought graphene and SiC into Al–Ni Alloy, discussing the effect of different content of SiC on the composite [15]. Samad et al. studied the copper composites reinforced with 5 wt.% TiC and different concentrations of GNP with machine learning enabled prediction [16]. Luo et al. prepared Cu matrix composites hybrid reinforced by GNPs and diamond particles, where the content of diamond remains 3.0 wt.% but the content of GNPs is changed [17]. Yehia et al. revealed the best amount of Al_2_O_3_-graphene hybrid nanocompsite in Al-5Ni-0.5 Mg alloy [18]. Alizadeh et al. characterized the wear and corrosion behavior of Al/Cu/MoS_2_/WC hybrid metal matrix composite synthesized via accumulative roll bonding process [19]. However, these studies still adopted single factor analysis, and the effect of low-dimensional material in multiple reinforcements was investigated independently to determine the optimal content of reinforcements.

Besides experimental studies, molecular dynamic (MD) simulations have been widely applied in investigating the strengthening mechanism of MMC. There are a lot of reports on the mechanical behaviors of MMC with single reinforcement at the early stage [20–22]. Nowadays, similar models are designed to study MMC with a particular structure. Das et al. estimated mechanical properties of aluminium-graphene core shell nano-composite under different conditions [23]. Ru et al. modelled the mechanical behavior of platinum-graphene nanocomposites for the purpose of specific engineering or biomedical applications [24]. Shen et al. fabricated heterogeneous CNTs/Al composites with a hierarchical structure by a parallel and series model [25]. Dai et al. employed models of MMC to compare atomic diffusion of diamond/Ni and multi-walled CNTs/Ni interfaces, but the model is built with one reinforcement at a time [26]. So, most of these models are just involved in MMC with single reinforcement.

To sum up, addition of multiple reinforcements is the hopeful way to further strength MMC, but the effects of multiple reinforcements have been not studied in depth, let alone the underlying strengthening mechanism on combination of different reinforcements in MMC. Sincerely, the investigation on the ratio in multiple reinforcements can not pave the way for underlying the combined strengthening mechanism. That is, this mechanism may not be revealed just by experimental methods, because it is quite difficult to control the combining form of reinforcements in experiment. Hence, we employ a MD simulation to change the location and state of reinforcements in MMC accurately, forming different combinations. Because of the very long length-diameter ratio of 1D nanotubes that is too long to be completely built in a limited box, only 2D nanoplate and 3D particle are chosen as reinforcements here. The studies mentioned above reveal that graphene is a very commonly used 2D reinforcing material, and diamond is selected as 3D reinforcement because it has the same atomic type with graphene. Hence, the structural combination of multiple reinforcements can be specifically studied, excluding the impact from different styles of pairwise interactions between reinforcement and matrix. The models with six combinations of reinforcements are designed carefully, and then the same sintering processes are operated on the designed models to obtain the proper structures of MMC. At last, the same tensile processes are applied on the sintered models. Thus, the effect of different combining form of reinforcement on the structures and mechanical properties of MMC can be discussed in detail.

## 2. Model methods

**Fig 1:**
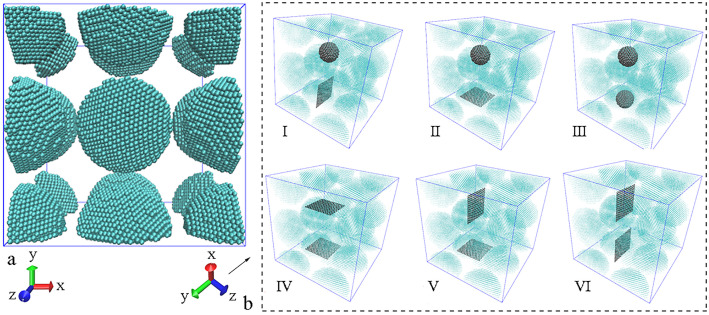
Initial models before sintering. (a) creating Al particles as matrix (b) inserting two reinforcements with different combining forms.

In order to build the models of Al matrix composite with different combining form of reinforcements, a simulation box with a side length of 12.8 nm is built at first, which contains eight Al spherical particles. These Al particles have a same radius of 28 Å and a same interval of 8 Å from each other, but the orientation of every Al nanoparticle is random. Because periodic boundary conditions (PBC) are applied in three dimensions of the box, a part of Al particles exit one end of the box and re-enter the other end, as shown in Fig 1a. Then, two kinds of reinforcements are inserted into the spaces between Al particles. Here, GNP serves as a 2D reinforced phase, while diamond particle acts a 3D reinforced phase, to study the enhancing effects of reinforcements in different combination. Since 2D nanomaterial is directional, the orientation of GNPs need to be taken into consideration, forming different combinations as shown from Ⅰ to Ⅵ in Fig 1b. In view of the powder sizes in experimental reports, the size of metal particles ranges from 18 to 58 µm, while the reinforcements have a sheet diameter of less than 10 µm or a particle size from 8 to 50 µm [1–3,10]. Here, the diameter of Al particles is 56 Å, so both graphene with the side length of 38.4 Å and diamond particle with the diameter of 24 Å are reasonable. Under these settings, the two reinforcements have almost the same surface area. Thus, the effect of reinforcements on composite can be comparable, since this effect on the motion and arrangement of metal atoms is brought by the interface [27]. Because of PBC condition, the arrangement of eight Al particles similar to body-centered cubic structure can form eight hollow cores. The central positions of two reinforcements are at the centers of two neighboring hollow cores. Thus, the initial structures for sintering the Al matrix composite have been established, and all the initial models are generated directly through LAMMPS software with the information above.

Because there are some spaces in the initial models to facilitate the insertion of reinforcements, every model will be relaxed for 100 picoseconds (ps) in NPT ensemble at 300 K and 1 atm before sintering, to make all the particles aggregate. To mimic the process of heating, holding and cooling in powder metallurgy, the temperature of relaxed models will rise from 300 K to 773 K and then reduce to 300 K, while the pressure of relaxed models will increase from 1 atm to 500 atm and then decease to 1 atm. Every process lasts for 300 ps and is conducted in NPT ensemble as well. This way to simulate the sintering process of Al matrix composite has been proved by some reports to be effective to gain sintered structures similar to experimental results such as the melting point and the density of composite [28,29].

To study the mechanical properties of sintered composite models, the quasi-static tensile process is operated under the NVE ensemble, and the temperature of sintered composite is maintained at 300 K by explicitly rescaling the velocities. The constant engineering strain rate is set to 0.002/ps, and the shape of simulation box in the tensile direction will be enlarged 20% in 100 ps. Through the same tensile process, every model will be stretched in X, Y and Z direction respectively. Furthermore, thermal conductivity of sintered models is also calculated to examine the connection with mechanical properties. In order to obtain a long enough structure for heat flow, the sintered model is replicated 20 times in one direction. The first unit serves as a hot source, while the eleventh unit end works as a cold source, as displayed in S1 Fig. Then, the heat will be added in the hot source at the rate of 5 eV/ps and be subtracted in the cold source at the same rate. When the model system achieves equilibrium, the thermal conductivity in this direction can be figured out according to the temperature difference between both ends of heat flow.

As GNP and diamond particle are used as the reinforcements in Al matrix composite, there are Al-Al, C-C and Al-C interaction between every two types of atoms, which take the style of *eam*, *airebo*, and *morse* potential separately. The *airebo* potential is very suitable for describing a system of carbon atoms, and the *morse* potential is assumed to be more reliable in interface. These pairwise potentials are generally used in the simulations about Al-C composite [28–31]. All the MD simulations are conducted through LAMMPS software (3 Mar 2020), with a time step of 1.0 femtosecond. The input scripts of sintering, stretching and heating are derived from examples form LAMMPS, and all the properties of sintered composite models are also calculated by LAMMPS, where a list of compute styles is available such as Voronoi volume, Centro-symmetry parameter (CSP), Common neighbor analysis (CNA) and so on. The structures of MD models are visualized by VMD software (1.9.3).

## 3. Results and discussions

### 3.1. The sintering process

**Fig 2:**
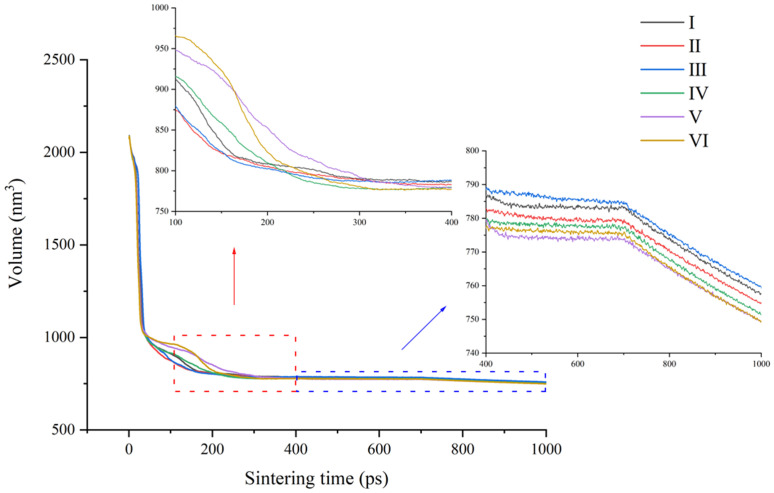
Bulk volumes of composite as a function of sintering time.

The evolution in bulk volume is traced to study the sintering process. The composite models are built in the same simulation box initially, so the volume of different models are the same at the beginning. After a relaxing time of 100 ps, a bigger volume is found in the composites with two pieces of GNPs, especially in model Ⅵ. Meanwhile, a smaller volume appears in the composite containing diamond particles, particularly in model Ⅱ and Ⅲ, as shown in the enlarged red area in Fig 2. It can be ascribed to the flexible property of GNP. If the two reinforcements is nearly in the same plane and one of them is GNP, GNP is easy to bend with all particles gathered, forming an enclosed space that cannot be fully filled with Al atoms under the low temperature and low pressure in relaxation. When the heating stage begins, the enclosed space seems to diminish gradually under acceleration of thermal motion. From the enlarged blue area in Fig 2, it can be easily founded that the bulk volumes of composites with two pieces of GNPs drop faster, even the smallest bulk volume is found in model Ⅴ at the end of heating stage, while the bulk volume of composite with two diamond particles is visibly larger. Then, a slight drop appears in all bulk volumes during the holding stage, but the order in bulk volume of composites remain unchanged, it implies the accomplishment of densification. The cold shrink in the cooling stage does not disturb the order in bulk volume as well, but the volume in model Ⅵ is almost as low as the volume in model Ⅴ. It should be noted that diamond particle itself occupy more space than GNP, enlarging the bulk volume of composite. However, comparison can be made between models that both contain one piece of GNP and a diamond particle (Ⅰ and Ⅱ), or in models that all contain two pieces of GNP (Ⅳ, Ⅴ and Ⅵ). After sintering, the volume of model Ⅰ or Ⅵ is distinctly larger. That is to say, the volume of the model where diamond is in the same plane as GNP could be bigger than that where diamond is out of the GNP plane, while the volume of the model where GNPs are parallel to each other is larger than that where GNPs are in a same plane. Thus, it is not difficult to be concluded that the different combining form of reinforcements plays an important role in promoting densification of metal matrix composite. It may be expected that a denser structure can be achieved by improving the combining form of reinforcements in the pressing process experimentally. If 2D nanoplate used to reinforce MMC, the parallel arrangement of 2D nanoplates should be avoided to achieve a compact composite. When both 2D nanoplate and 3D particles are applied in MMC, the spatial combination where 3D particle is out of the plane of 2D nanoplate can be more conducive to densification.

### 3.2. The sintered structure

**Fig 3:**
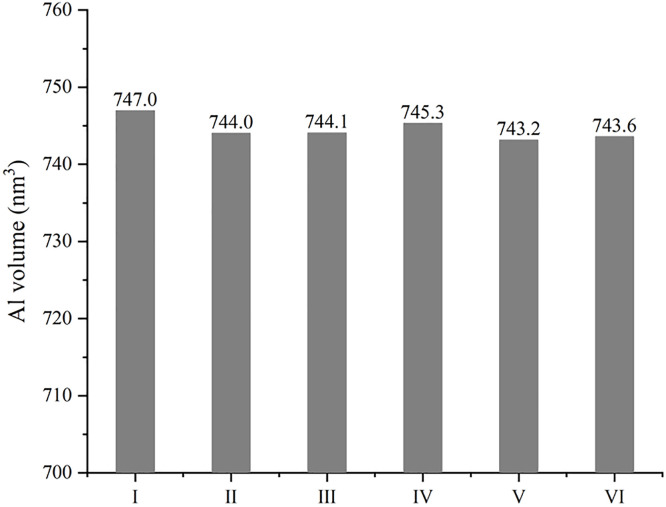
Al volumes of composites with different combining forms of reinforcements.

To make the sintered structure clearer, Al volume in those models is calculated separately based on the Voronoi tessellation of the atoms. As listed in Fig 3, there is a small difference in Al volume among the sintered models. Every model has the same quantity of Al atoms (46176), and the number of carbon atoms in one diamond particle or GNP is 1279 or 558. Therefore, the density of Al in composite can be calculated, which ranges from 2.79 to 2.81 g/cc, and this density is consistent with the experimental results [29]. As far as the models here, there seems to be no significant difference on densification between different kinds of reinforcements, Al volume in model containing two diamond particles (Ⅲ) is very close to Al volume in model containing two GNPs (Ⅴ and Ⅵ). However, Al volume in model Ⅰ is bigger than that in model Ⅱ, while Al volume in model Ⅳ is bigger than that in model Ⅴ or Ⅵ. This result is coincided with the conclusion from the sintering process, and it means that the combining form of reinforcements can definitely affect the aggregation of metal particles, with making a difference in volume. Overall, the combination where 3D particle is out of the plane of 2D nanoplate is more beneficial to a compact structure, and more 2D nanoplates can make the composite denser unless they are parallel to each other.

**Fig 4:**
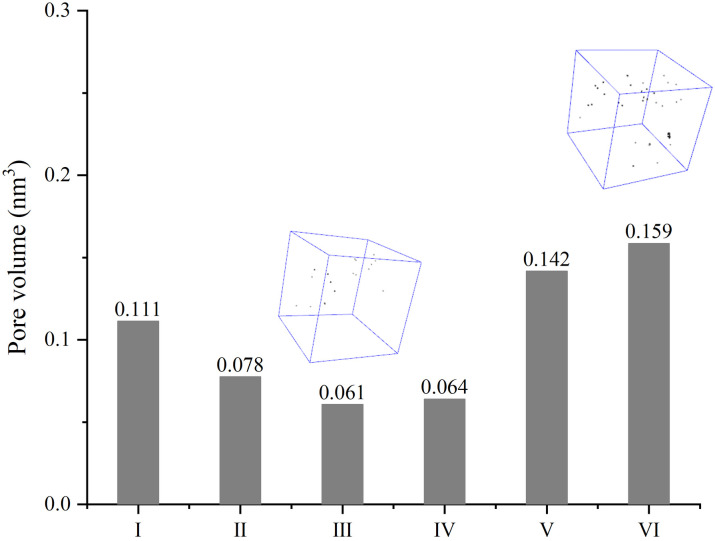
Pore volumes of composites with different combining forms of reinforcements.

To account for the difference in Al volumes, the nanopores are figured out as well. The void in composite where the diameter is larger than 4 Å here is considered as the nanopore. At this scale, the pore volumes are far less than Al volumes. The porous configurations in composite reveal that the dense texture can be produced in all sintered models, where only a small number of pores are evenly distributed in the composite structure, even with the highest porosity. Strictly speaking, Fig 4 could indicate that GNP is likely to bring an increase in pore volume to some extent, but GNPs that are parallel to each other may avoid formation of more pores. Therefore, it can be also supposed that the combination form of reinforcements has a certain influence on nanopores, although the gap in pore volume is less than 0.1 nm^3^. However, that gap can not account for the disparity in Al volume, and the tendency in pores is not exactly the same as the change in metal volume. The structure of model Ⅳ is identified as a high Al volume but a low pore volume. Thus, a different conclusion can be drawn from model Ⅰ, Ⅴ and Ⅵ in Fig 4. More nanopores in MMC will appear when another reinforcement is in the same plane as the 2D nanoplate.

**Fig 5:**
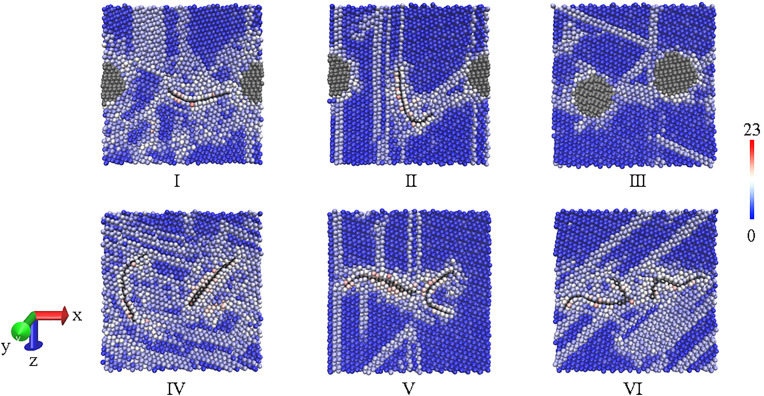
Inner structure of sintered composites, where Al atoms are colored in CSP values and reinforcements are tinted in black.

**Fig 6:**
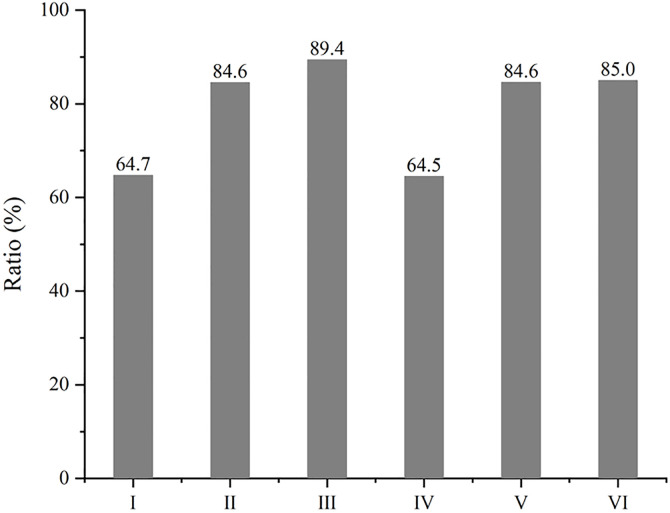
The ratio of arranged Al atoms in composites with different combining forms of reinforcements.

To gain insight in the inner structure of sintered models, Al atoms are colored in the CSP values, and the sectional view passing through the center of reinforcements are visualized. The blue balls stand for atoms with perfect lattice, while the graduated color implies the increase in local lattice disorder from local defect to free surface. Fig 5 reveals that the arrangement of Al atoms can be visibly disturbed by reinforcements. With a shrink in the composite after sintering, GNPs in all composites have various degrees of bending, but the locations and states of reinforcements can still exhibit the different combination forms visually. Most of Al atoms surrounding diamond are tinted white, and even a few Al atoms beside GNP are printed red. It is in agreement with the experimental report, which argued that graphene could affect the motion and arrangement of metal atoms heavily [27]. Thus, GNP seems to bring a greater influence than diamond on adjacent atoms. This grain refinement has been reported by many studies [29,30]. Nevertheless, the single sectional view may not capture the full effect of the combination form of reinforcements. Therefore, CNA analysis is operated on sintered models, both f.c.c (face-centered cubic) and h.c.p (hexagonal close-packed) atoms are counted as arranged atoms in Fig 6. There are less arranged atoms in both model Ⅰ and Ⅳ, and the others have more atoms with perfect lattice. This is coincided with the tendency in Al volume, where Al volume in model Ⅰ or Ⅳ is relatively bigger. It means that the more compact structure may be derived from the rise in the quantity of arranged atoms.

### 3.3. The tensile process

**Fig 7:**
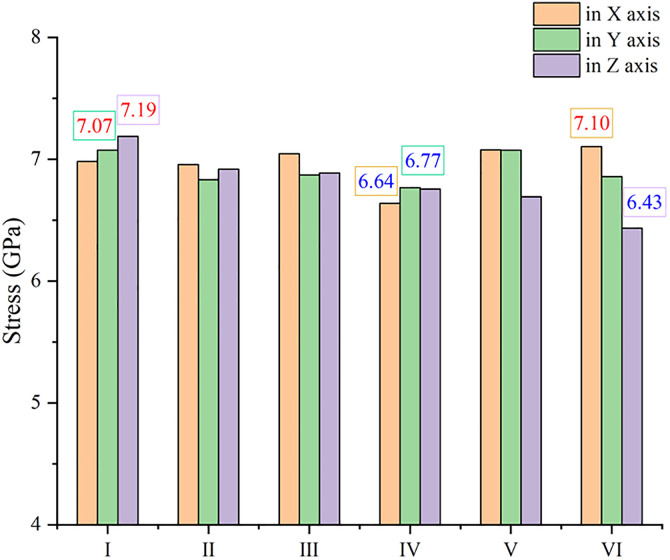
The tensile strength of composite with different combining forms of reinforcements along different axis.

The sintering process above aims at producing the sintered structure with different combining form of reinforcements, and then the mechanical property of that can be investigated through the tensile process in different direction. The tensile strength of sintered models is displayed in Fig 7, where the maximum or the minimum value in every direction is labelled. Unexpectedly, the composite with two GNPs seems to be weaker, only the highest tensile result along X axis appears in model Ⅵ. In fact, this conclusion is very consistent with the directionality of GNP. If the tensile results are considered with inner structures in Fig 5, it can be easily found the best state of GNP is that the load is just applied along the plane of GNP, especially along the plane where two GNPs lie with one in front of the other (model Ⅵ). On the contrary, GNPs may not work well if the direction of the load is perpendicular to the plane of GNP, so the lowest tensile result along Z axis appears in model Ⅵ, too. However, when GNPs are parallel to each other, it is easy for tensile force to make them slide away from each other, so mechanical property of model Ⅳ is very weak, and the minimum values along both X and Y axis are observed here. Overall, model Ⅰ has the best performance in tension with the maximum values along both Y and Z axis, and the tensile strength of that along X axis is not low as well.

**Fig 8:**
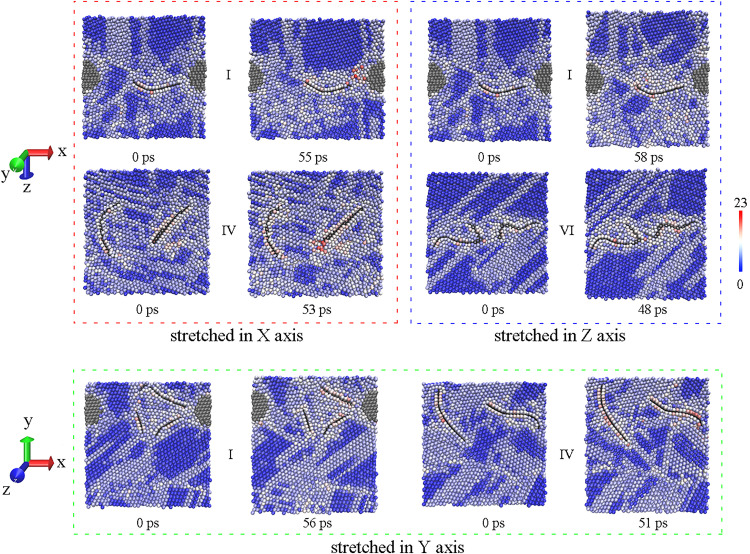
Comparisons on inner structure between model Ⅰ and the weakest, the left is before stretching and the right is at maximum stress in every block.

Atomic configures of inner structures are detailed to compare model Ⅰ having the best performance and the weakest structure. As shown in Fig 8, there are visible voids beside GNPs, since red atoms stand for atoms at surface. However, the crack is not formed along GNP with the propagation of dislocations in the red block of Fig 8, the crack arise between diamond and GNP in model Ⅰ, it means that GNP can be effective to transfer load when the load is along GNP plane. Likewise, the section of model Ⅰ in green block has not changed much at maximum stress. Therefore, the mechanical property of model Ⅰ along both X and Y axis should be fine. On the contrary, in model Ⅳ where GNPs are parallel, the crack appears at the end of GNP in both red and green block, resulting in a poor result. It is worth mentioning that model Ⅵ is weakest in Z axis as drawn in blue block, and this direction is largely perpendicular to the GNPs in model Ⅵ. It implies that GNP may have no positive effect when the load is normal to GNP plane. Similarly, the tensile result of model Ⅴ in Z axis is not good as well. Whereas, model Ⅰ having the best performance along Z axis can only be ascribed to the combination of GNP and diamond, the location and state of both reinforcements are little changed at maximum stress, accompanied by accumulation of dislocations. Likely, model Ⅱ does well in X axis, although the load direction is vertical to GNP as well. That is to say, diamond in combination can make up for the low mechanical performance vertical to GNP.

**Fig 9:**
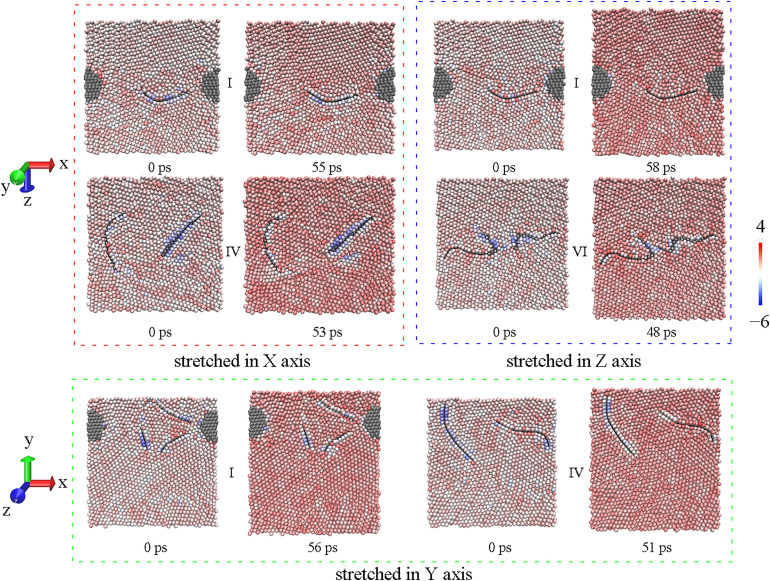
Stress distribution (unit: × 10^6^ bar·Å^3^) of Al atoms in model Ⅰ and the weakest, the left is before stretching and the right is at maximum stress in every block.

In order to provide the details in the local mechanisms of load transfer, the stress distribution of Al atoms in the composites is colored by per-atoms stress in Fig 9, which corresponds to inner structures in Fig 8. When the load is along the GNP plane (such as model Ⅰ in red block), quite a few Al atoms surrounding GNP bear compressive stress before stretching, and can keep this state even when the overall stress is at maximum. The Al atoms surrounding GNP is inclined to form the close-packed plane owing to the honeycomb lattice of GNP, which is a little smaller than the lattice of perfect Al lattice. The high elastic modulus property of graphene restricts the deformation along the GNP plane. When the load is vertical to GNP plane (such as model Ⅰ in blue block), the stress distribution of Al atoms surrounding GNP is not much different from that far away from GNP. That is to say, graphene can play a greater role in load transfer along the GNP plane. Thus, diamond particle may have a better performance under the load normal to graphene, compensating the directional constraint of GNP.

**Fig 10:**
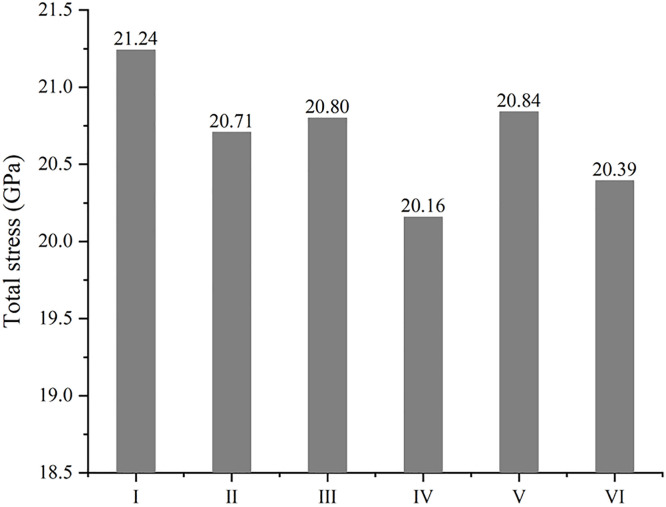
The total tensile strength of composites with different combining forms of reinforcements.

Because of anisotropy in tensile property of models above, the overall performance is simply estimated by adding up the three values in different direction (Fig 10). Model Ⅰ is clearly the highest in total tensile strength where diamond is in the same plane as GNP, and model Ⅳ gets the lowest point with GNPs parallel to each other. It is worth noting that the indirect effect of reinforcements such as grain refinement may not play a role at this atomic scale, owing to inverse hall-patch relation [32]. That is, the advantage of model Ⅰ may be greater in view of the lower ratio of arranged atoms in Fig 6. It is also the reason models with two GNPs have not shown the superiority over models with two diamond particles here. To sum up, it can be assumed that the combining form of reinforcements makes big difference in mechanical property of MMC, and the structure like model Ⅰ enhanced by 2D nanoplate and 3D particle in a same plane can give the best performance in strength for MMC. It is worthy mentioned that there is a big difference in some experimental reports on the mechanical property of graphene-Al composite, where the reinforced results on strength fluctuate from 30% to more than 120% [7,10,15]. This disparity may be produced by different preparing processes, which form different microstructures. This simulation with different combination of reinforcements could account for the discrepant experimental results very well. In order to study the connection between thermal and mechanical behavior in metallic composites, the thermal conductivity of every model in each direction is calculated by MD simulation as well. As shown in S2 Fig, graphene brings the directionality in thermal conductivity, and only the composite with two diamond particles (model III) has almost the same result in each direction. Overall, model Ⅰ has the lowest thermal conductivity that is corresponding to the highest strength, although a diamond particle has much more atoms than a GNP here. Because of the wide difference in particle size and grain size, the results of thermal conductivity (from 217 to 311 W/mK) are lower than experimental reports of Al-diamond composite (from 313 to 475 W/mK) [17,33]. However, the connection between thermal and mechanical behavior is the same as that in experiment, where thermal conductivity increases with a drop in mechanical property [34].

**Fig 11:**
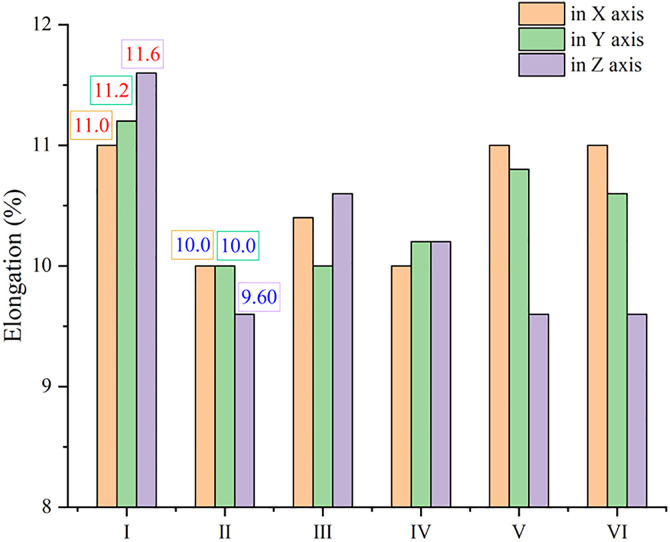
The elongation of composites with different combining forms of reinforcements at maximum stress along different axis.

**Fig 12:**
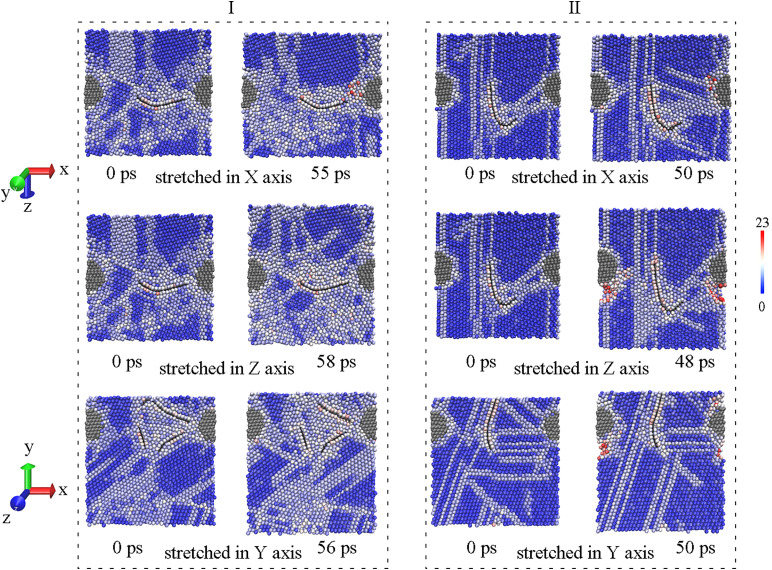
Comparisons on inner structure between model Ⅰ and Ⅱ, the left is before stretching and the right is at maximum elongation in every block.

**Fig 13:**
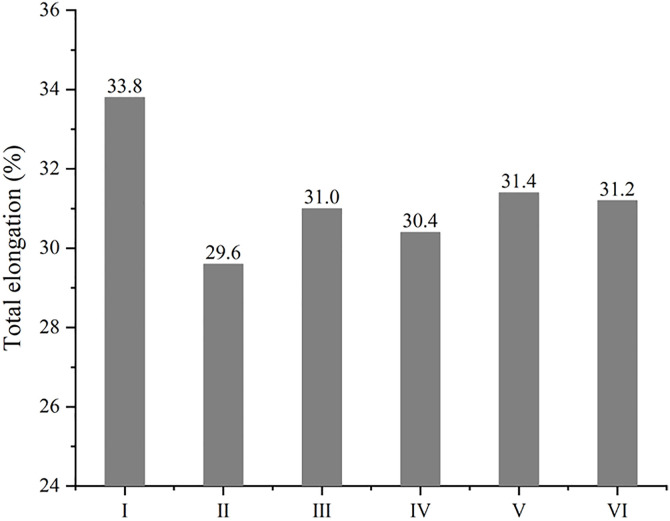
The total elongation of composites with different combining forms of reinforcements at maximum stress.

Besides the advantage in strength, model Ⅰ is excellent in ductility as well. Fig 11 depicts the elongation at maximum stress along different axis, and all the highest values of elongation are observed in model Ⅰ. As expected, GNP can improve the ductility of MMC when stretched along GNP plane, just like model Ⅵ that does a little better in X axis than in Y axis and does worst in Z axis. However, a piece of GNP seems to be enough to improve the ductility along GNP plane, because there is a small gap among model Ⅰ, Ⅴ and Ⅵ in X axis. Therefore, GNP is deemed to be beneficial to ductility of MMC, and this conclusion are supported by many experimental reports [10,15]. However, model Ⅳ also has two GNPs, giving rise to a short elongation in every direction. It can be attributed to the low strength, which leads to an early fracture. The disparity in effects of GNP on ductility is also in accord with experimental reports. Except for the favorable reports mentioned above, a drop in elongation was found after addition of graphene [35,36], and this simulation could explain both a rise and a drop in ductility of metal/graphene composite. Unexpectedly, all the lowest values of elongation are obtained in model Ⅱ, which also contains a piece of GNP. Based on Fig 12, clear cracks appear near the diamond in model Ⅱ, it can be safely believed that the role of GNP in that combination does not match with diamond particle out of GNP plane, and stiffness of diamond limits the elongation of MMC, when stretched in Y and Z axis. If stretched in X axis, GNP in model Ⅱ is not conducive to elongation either, because the load is normal to GNP. More emphasis should be placed on model Ⅰ stretched in Z axis that gets the longest value, though the load is not along GNP plane as well. As displayed in Fig 12, this combination form of model Ⅰ can suffer from accumulation of more dislocations before the occurrence of crack, giving the best performance in strength at the same time, and different combination forms of GNPs here can explain this disparity. In brief, model Ⅰ is the most clearly in favor of ductility too, serving as the best combination form of reinforcements in MMC, according to Fig 13. The fracture morphologies of model Ⅰ and Ⅱ in the sectional views across cracks are visualized in S3 Fig. All fractures occur far away from GNP, except for model Ⅰ stretched in Z axis. It further confirms that diamond in model Ⅰ can compensate the weakness of graphene along GNP plane.

## 4. Conclusions

Models of Al matrix composite enhanced by two kinds of reinforcements are built here to study the effect of different combination form of reinforcements. Diamond and GNP are selected as 3D and 2D reinforcement, forming six combinations. By modelling the sintering process, compact structures of MMC are achieved. According to the curves of bulk volumes and Al volume in sintered composite, the denser structure can be formed under the combination, where 2D reinforcements those are not parallel to each other or 3D particle is out of the plane of 2D nanoplate. More nanopores are detected when another reinforcement exists in the same plane of 2D reinforcement. However, the porosity in every model is not high. So the difference in volume should be ascribed to the change in the number of arranged atoms, based on CNA analysis. The same tensile processes are conducted on the sintered composites, to investigate the combined strengthening mechanism in depth. The model enhanced by 2D nanoplate and 3D particle in a same plane is confirmed to have the best performance both in strength and in ductility. CSP configurations indicate that GNP can be effective to transfer load along the GNP plane, and diamond can make up for the low mechanical property perpendicular to GNP. However, the composite reinforced with parallel GNPs is weakest in strength and the composite where diamond is out of the plane of GNP is lowest in ductility. In consideration of the low ratio of arranged atoms in composites, which may further improve mechanical property owing to grain refinement, the best combination form of reinforcements is believed to be the model enhanced by 2D nanoplate and 3D particle in a same plane.

## Supporting information

S1 Fig The model with replicated units for thermal conductivity. (TIF)

S2 Fig The thermal conductivities of composite with different combining forms of reinforcements along different axis. (TIF)

S3 Fig The fracture morphologies of model Ⅰ and Ⅱ in the sectional views across cracks. (TIF)

S4 Table Values of bulk volumes with respect to sintering time in every sintering process. (PDF)

S5 Table Values of stress with respect to strain in every tensile process. (PDF)

S6 Table Values in structural and mechanical analysis on sintered models. (DOCX)

## References

[1] Sharma P, Sharma S, Khanduja D. Production and some properties of Si3N4 reinforced aluminium alloy composites. Journal of Asian Ceramic Societies. 2015;3(3):352–9. doi: 10.1016/j.jascer.2015.07.002

[2] Veeresh Kumar GB, Panigrahy PP, Nithika S, R P, Rao CSP. Assessment of mechanical and tribological characteristics of silicon nitride reinforced aluminum metal matrix composites. Composites Part B: Engineering. 2019;175:107138.

[3] Wang Z-G, Li C-P, Wang H-Y, Zhu X, Wu M, Jiang Q-C. Effect of nano-SiC content on mechanical properties of SiC/2014Al composites fabricated by powder metallurgy combined with hot extrusion. Powder Metallurgy. 2016;4:59. 10.3390/ma9120964 PMC5457012 28774083

[4] Zhang J-D, Zhang L, Ma HZ, Li N. Effects of ZrC content on microstructures and properties of ZrC/AlCoCrFeNi high-entropy alloys composites. Journal of Alloys and Compounds. 2025;1010:178308. doi: 10.1016/j.jallcom.2024.178308

[5] Jin P, Zhou J, Zhou J, Liu Y, Sun Q. Contribution made by double-sized TiC particles addition to the ductility–strength synergy in wire and arc additively manufactured Al–Cu alloys. Composites Part B: Engineering. 2024;268:111078. doi: 10.1016/j.compositesb.2023.111078

[6] Hou J, Qian B, Zhu Z, Zou S, Li G, Zhu Q, et al. A mechanical strong yet ductile CoCrNi/Cr2B composite enabled by in-situ formed borides during laser powder bed fusion. Composites Part B: Engineering. 2024;278:111428.

[7] Zhou S, Zhang W, Liu M, Ren W, Yang Y, Zhou Q, et al. Microstructure evolution and tensile properties tailoring of graphene nanoplatelets/nickel composites fabricated by two-step 3D vibration milling. Journal of Alloys and Compounds. 2022;918:165676.

[8] Liu C, Jiang X, Sun H, Liu T, Wu Z, Yang L. Preparation of graphene film reinforced CoCrFeNiMn high-entropy alloy matrix composites with strength-plasticity synergy via flake powder metallurgy method. Journal of Materials Research and Technology. 2023;27:7614–26.

[9] Gao Y, Zou J, Wang X, Wang X, Yang J, Wang H. Microstructure and Mechanical Performance of Graphene Nanosheets Reinforced Nickel-Based Superalloy FGH95 Composite. Nanomaterials (Basel). 2020;10(1):100. doi: 10.3390/nano10010100 31947832 PMC7022809

[10] Lou S, Cheng B, Liu Y. Influence of extrusion temperature on the microstructure and mechanical properties of a 0.5 wt.% graphene nanoplatelet-reinforced aluminum composite. J Materi Eng Perform. 2023;32:9344–56.

[11] Tian D, Liu Y, Yu J, Zhao Q, Tao J, Wu Z, et al. A Study of Silver Decoration on Carbon Nanotubes via Ultrasonic Chemical Synthesis and Their Reinforced Copper Matrix Composites. Nanomaterials. 2023;13:887. 36903767 10.3390/nano13050887 PMC10005354

[12] Kim D, Hirayama Y, Liu Z, Takagi K, Kobashi M. Fabrication of Al-CNT composite with high hardness and electrical conductivity by controlling Al4C3 formation. Journal of Alloys and Compounds. 2023;942:169102. doi: 10.1016/j.jallcom.2023.169102

[13] Cao L, Chen B, Wan J, Shen J, Kondoh K, Li S, et al. Simultaneously improving strength and ductility of carbon nanotube (CNT)-reinforced aluminum matrix composites by embedding CNTs inside matrix grains. Composites Part B: Engineering. 2025;296:112240.

[14] Mustu M, Demir B, Aydin F. An investigation of mechanical and wear performance of TiB2/GNPs-reinforced ZK60 Mg matrix composites fabricated via powder metallurgy. J Materi Eng Perform. 2023;32:3527–41.

[15] Elkady OA, Yehia HM, Ibrahim AA, Elhabak AM, Elsayed ElsayedM, Mahdy AA. Direct Observation of Induced Graphene and SiC Strengthening in Al–Ni Alloy via the Hot Pressing Technique. Crystals. 2021;11(9):1142. doi: 10.3390/cryst11091142

[16] Samad A, Arif S, Ansari S, Muaz M, Mohsin M, Khan AU, et al. Machine learning enabled prediction of tribological properties of Cu-TiC-GNP nanocomposites synthesized by electric resistance sintering: A comparison with RSM, Journal of Materials Research and Technology, 2024;28:2290–312.

[17] Fang L, Xiaosong J, Hongliang S, Defeng M, Yali Z, Rui S, et al. Microstructures, mechanical and thermal properties of diamonds and graphene hybrid reinforced laminated Cu matrix composites by vacuum hot pressing. Vacuum. 2023;207:111610.

[18] Yehia HM, Nyanor P, Daoush WM. Characterization of Al-5Ni-0.5Mg/x (Al2O3-GNs) nanocomposites manufactured via hot pressing technique. Materials Characterization. 2022;191:112139.

[19] Alizadeh M, Karimi A, Pashangeh S, Ostovari moghaddam A. Characterization of wear and corrosion behavior of Al/Cu/MoS2/WC hybrid metal matrix composite fabricated via accumulative roll bonding process. Journal of Materials Research and Technology. 2025;35:4647–59.

[20] Srivastava AK, Pathak VK, Singh R, Dikshit MK. Stress-strain behaviour of graphene reinforced aluminum nanocomposite under compressive loading using molecular dynamics. Materials Today: Proceedings. 2021;44:4521–5.

[21] Han RQ, Song HY, An MR. Atomic simulation of interaction mechanism between dislocation and graphene in graphene/aluminum composites. Computational Materials Science. 2021;197:110604. doi: 10.1016/j.commatsci.2021.110604

[22] Huo S, Xie L, Xiang J. Atomic-level study on mechanical properties and strengthening mechanisms of Al/SiC nano-composites. Appl Phys A. 2018;124:209.

[23] Das DK, Kumar B. Mechanical properties of aluminium-graphene core shell nano-composite: A molecular dynamics simulation study. Diamond and Related Materials. 2025;152:111981. doi: 10.1016/j.diamond.2025.111981

[24] Ru Y, Basem A, Hussein RA, Singh NSS, Al-Bahrani M, Salahshour S, et al. Modeling the mechanical behavior of platinum-graphene nanocomposites prepared via powder metallurgy at various initial temperatures and pressures. International Communications in Heat and Mass Transfer. 2025;163:108727. doi: 10.1016/j.icheatmasstransfer.2025.108727

[25] Shen M, Hao Z, Song J, An M, Ying T, Xue X, et al. Architectural and component design of CNTs/Al hierarchical composite for enhanced mechanical/thermal properties. Journal of Materials Research and Technology. 2024;30:120–33. doi: 10.1016/j.jmrt.2024.03.062

[26] Dai J, Wei D, Wang Z, Si L, Cao S, Liu H, et al. Comparative research on atomic diffusion of diamond/Ni and MWCNTs/Ni interfaces with molecular dynamics and experimental methods. Ceramics International. 2025;51:5434–50.

[27] Shin SE, Choi HJ, Hwang JY, Bae DH. Strengthening behavior of carbon/metal nanocomposites. Sci Rep. 2015;5:16114. doi: 10.1038/srep16114 26542897 PMC4635460

[28] He HP, Rong Y, Zhang L. Molecular dynamics studies on the sintering and mechanical behaviors of graphene nano-platelet reinforced aluminum matrix composites. Model Simul Mater Sci Eng. 2019;27:065006.

[29] Zhu Y, Li N, Zhang L, Zhang J, Niu L, Li W, et al. Atomistic Investigation of the Effects of Different Reinforcements on Al Matrix Composite. Metals. 2022;12(8):1252. doi: 10.3390/met12081252

[30] Zhu Y, Sui C, Li N, Sun L, Li S. Large-Scale Atomistic Simulation of Sintering Process and Mechanical Properties of Al Matrix Composite with Different Reinforcements. Metals. 2024;14:1312.

[31] Zhu Y, Li N, Li W, Niu L, Li Z. Atomistic Study on the Sintering Process and the Strengthening Mechanism of Al-Graphene System. Materials (Basel). 2022;15(7):2644. doi: 10.3390/ma15072644 35407976 PMC9000460

[32] Conrad H, Narayan J. On the grain size softening in nanocrystalline materials. Scr Mater. 2000;42:1025–30.

[33] Wei N, Zhou C, Li Z, Ou B, Zhao K, Yu P, et al. Thermal conductivity of aluminum/graphene metal-matrix composites: From the thermal boundary conductance to thermal regulation. Materials Today Communications. 2022;30:103147.

[34] Tan Z, Chen Z, Fan G, Ji G, Zhang J, Xu R, et al. Effect of particle size on the thermal and mechanical properties of aluminum composites reinforced with SiC and diamond. Materials & Design. 2016;90;845–51.

[35] Yang W, Zhao Q, Xin L, Qiao J, Zou J, Shao P, et al. Microstructure and mechanical properties of graphene nanoplates reinforced pure Al matrix composites prepared by pressure infiltration method. J. Alloys Compd. 2018;732:748–58.

[36] Wang J, Guo L, Lin W, Chen J, Liu C, Chen S, et al. Effect of the graphene content on the microstructures and properties of graphene/aluminum composites. New Carbon Materials. 2019;34(3):275–85. doi: 10.1016/s1872-5805(19)60016-8

