# Supplementary figures and images for: Atomistic study on mechanical properties of Al matrix composite with different combining forms of reinforcements
Source: PLoS One. 2025 Aug 11;20(8):e0329889. doi: 10.1371/journal.pone.0329889 (PMC12338809; doi:10.1371/journal.pone.0329889)

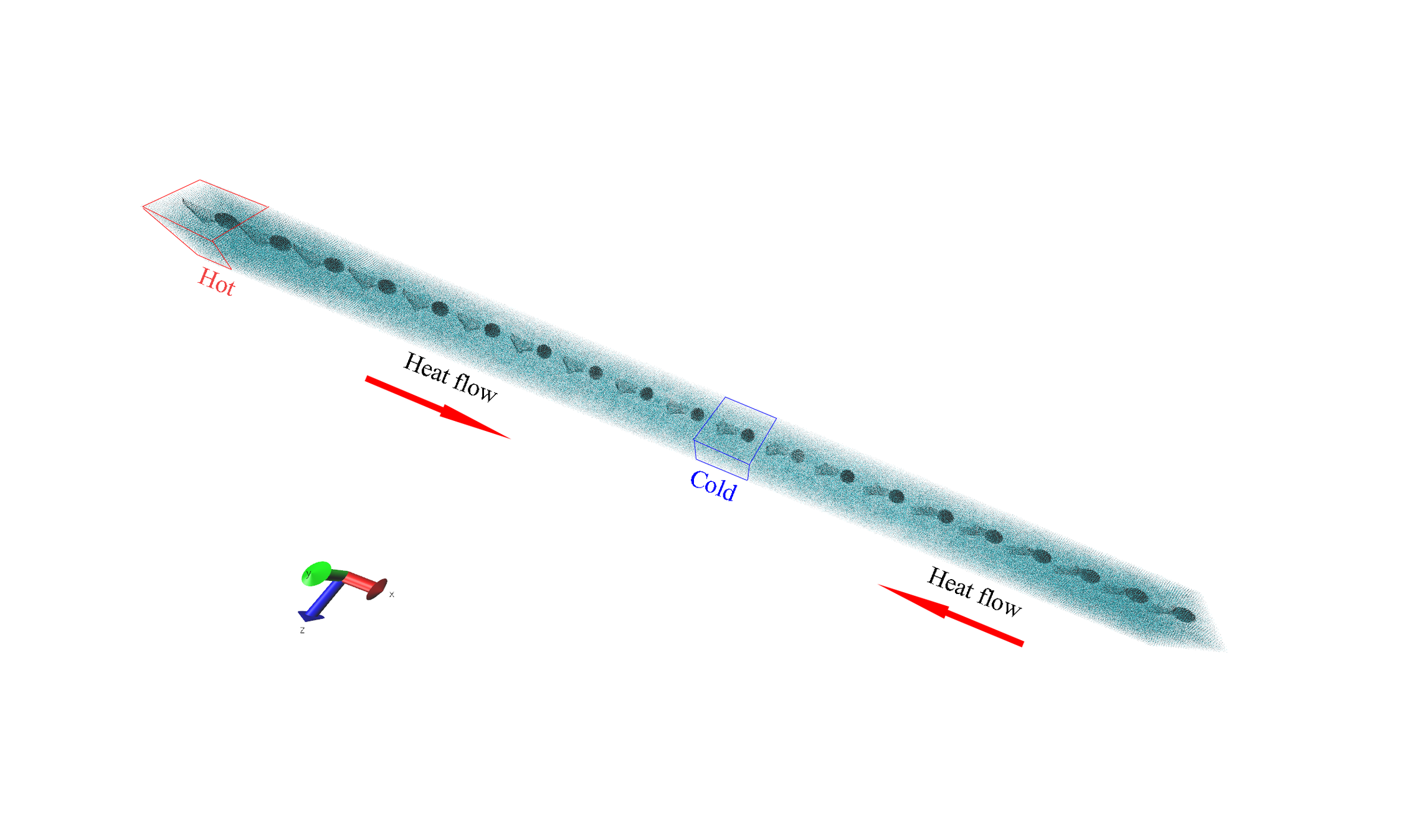

Supplement: S1 Fig — (TIF) [file pone.0329889.s001.tif]

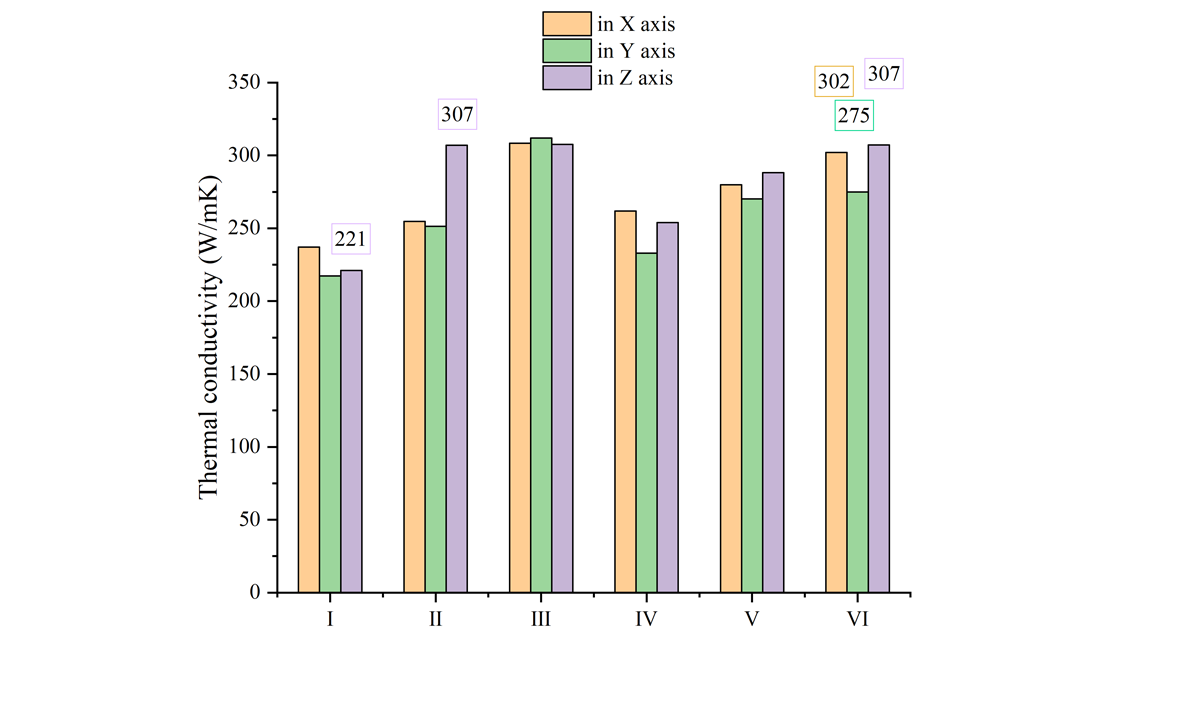

Supplement: S2 Fig — (TIF) [file pone.0329889.s002.tif]

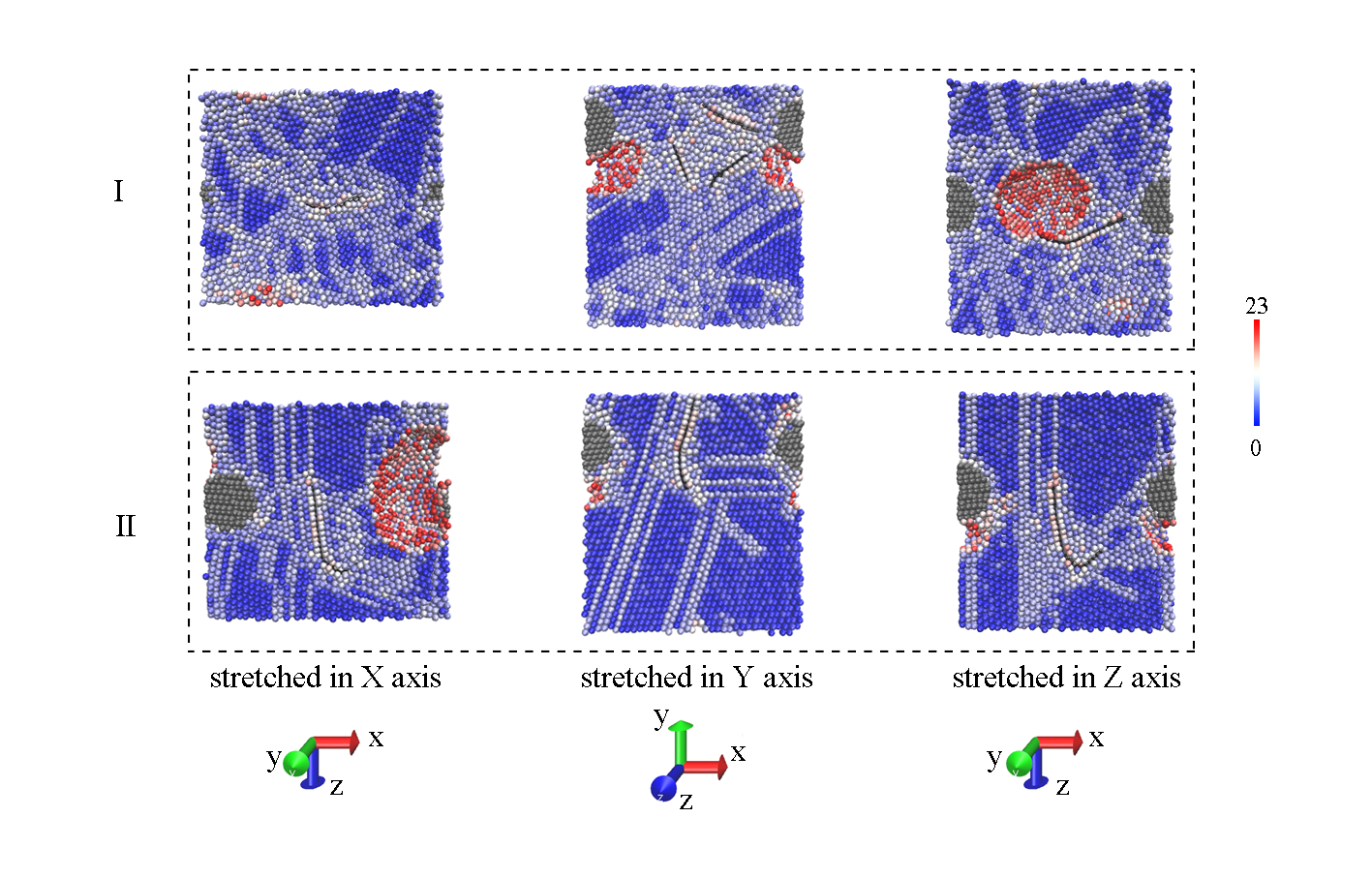

Supplement: S3 Fig — (TIF) [file pone.0329889.s003.tif]
