# Supplementary material for: Atomistic study on mechanical properties of Al matrix composite with different combining forms of reinforcements
Source: PLoS One. 2025 Aug 11;20(8):e0329889. doi: 10.1371/journal.pone.0329889 (PMC12338809; doi:10.1371/journal.pone.0329889)
Supplement: S4 Table — (PDF) [file pone.0329889.s004.pdf]

Sintering time /(ps)	Bulk volumes/(nm³)					
	I	II	III	IV	V	VI
0.25	2075.21	2075.21	2075.21	2075.21	2075.21	2075.21
1.00	2090.69	2089.16	2091.37	2089.64	2089.41	2089.42
2.00	2070.88	2071.04	2069.99	2071.62	2071.60	2070.29
3.00	2047.59	2047.54	2046.53	2049.62	2049.60	2048.11
4.00	2034.05	2033.02	2032.33	2032.20	2032.28	2031.53
5.00	2022.95	2018.80	2021.09	2015.08	2016.47	2014.74
6.00	2009.89	2007.06	2009.27	1997.57	2000.62	1998.32
7.00	2000.73	2000.25	2001.11	1984.37	1988.17	1985.35
8.00	1991.86	1992.95	1992.47	1971.18	1979.05	1976.63
9.00	1985.67	1985.92	1984.24	1960.57	1971.39	1970.38
10.00	1980.98	1977.85	1977.23	1957.37	1964.63	1959.56
11.00	1978.44	1969.55	1970.23	1951.37	1953.94	1943.62
12.00	1973.18	1962.21	1964.30	1941.77	1941.35	1930.44
13.00	1962.77	1952.96	1956.97	1930.21	1929.67	1918.94
14.00	1950.39	1944.57	1950.71	1918.95	1914.73	1903.60
15.00	1933.19	1937.15	1943.54	1899.29	1894.00	1881.00
16.00	1912.34	1933.89	1937.21	1873.72	1872.49	1849.61
17.00	1889.48	1930.26	1931.23	1846.96	1848.14	1803.18
18.00	1860.16	1927.17	1923.87	1821.54	1821.14	1732.33
19.00	1816.70	1921.32	1915.73	1777.89	1777.73	1643.72
20.00	1748.44	1912.10	1906.94	1693.68	1689.26	1551.18
21.00	1658.61	1897.75	1893.43	1577.33	1574.70	1459.08
22.00	1559.73	1875.58	1871.84	1469.61	1474.33	1385.74
23.00	1469.25	1827.00	1825.82	1403.86	1398.94	1333.98
24.00	1408.33	1742.47	1745.11	1356.04	1348.09	1285.05
25.00	1373.06	1638.63	1640.17	1306.90	1300.85	1224.97
26.00	1333.57	1536.17	1533.17	1260.15	1246.95	1155.23
27.00	1285.05	1448.39	1446.78	1201.86	1185.85	1107.86
28.00	1226.02	1391.93	1397.76	1144.01	1130.19	1079.92
29.00	1165.77	1348.67	1363.82	1101.52	1095.04	1066.91
30.00	1115.09	1301.63	1321.81	1076.66	1073.58	1054.86
31.00	1082.63	1246.91	1279.75	1061.88	1065.06	1044.91
32.00	1064.93	1186.92	1228.19	1051.73	1055.35	1036.01
33.00	1050.44	1132.73	1173.78	1041.33	1047.60	1030.11
34.00	1040.37	1086.55	1120.49	1034.52	1041.47	1025.47
35.00	1031.09	1055.33	1078.00	1028.56	1037.49	1021.89
36.00	1024.33	1036.79	1054.22	1022.06	1035.76	1019.27
37.00	1017.56	1027.01	1041.35	1018.01	1033.91	1017.74
38.00	1012.29	1020.03	1031.21	1013.21	1029.53	1015.91
39.00	1009.14	1014.27	1023.79	1008.21	1027.38	1013.17
40.00	1003.44	1007.09	1018.52	1004.67	1024.85	1011.74
41.00	999.43	1001.53	1014.09	1000.49	1020.70	1009.04
42.00	996.65	996.18	1010.00	996.01	1018.91	1008.50
43.00	993.19	990.92	1004.81	992.71	1014.59	1007.73
44.00	987.64	985.73	1000.78	988.07	1010.73	1007.63
45.00	986.35	982.56	997.50	985.61	1010.23	1006.64
46.00	984.61	980.18	994.05	981.47	1006.60	1004.68
47.00	981.27	976.79	991.71	978.89	1003.85	1003.74
48.00	978.56	973.89	987.77	977.78	1000.48	1002.54
49.00	976.47	969.52	983.93	976.08	997.97	1000.73

50.00	975.30	967.93	981.24	973.83	997.31	999.07
51.00	973.14	963.19	979.08	971.47	995.65	999.75
52.00	971.47	960.84	976.35	968.96	994.72	997.52
53.00	969.20	957.47	973.08	967.06	993.22	996.80
54.00	967.41	956.38	970.70	964.39	991.21	994.48
55.00	964.63	952.54	968.94	963.46	989.73	994.92
56.00	962.70	949.97	967.12	963.46	987.56	993.69
57.00	960.23	948.61	965.82	961.52	987.44	991.60
58.00	958.93	945.78	963.71	960.10	985.80	991.75
59.00	957.57	943.80	961.55	959.35	985.30	989.86
60.00	956.05	941.10	960.09	958.19	984.47	988.77
61.00	955.38	940.06	958.94	956.21	983.25	988.60
62.00	954.01	936.57	956.49	952.97	983.60	985.75
63.00	952.39	935.15	953.06	952.17	982.49	984.45
64.00	951.90	933.88	951.78	948.76	980.72	984.57
65.00	951.51	931.53	949.69	947.41	980.30	984.34
66.00	947.98	929.66	948.26	945.83	979.12	982.66
67.00	946.83	926.32	944.51	943.37	977.30	981.79
68.00	945.45	924.46	942.96	942.21	977.25	980.42
69.00	944.03	921.66	940.97	941.23	977.36	978.76
70.00	943.32	918.20	938.62	939.56	975.79	977.58
71.00	942.71	917.26	936.51	939.23	974.24	978.44
72.00	942.41	915.80	934.40	938.01	973.19	977.79
73.00	941.21	912.49	933.22	937.08	971.82	977.58
74.00	940.26	910.93	930.70	936.89	970.85	976.12
75.00	938.54	909.62	928.15	935.87	970.56	975.98
76.00	938.50	907.45	926.82	934.46	970.44	974.97
77.00	937.51	905.56	924.56	934.40	968.81	975.06
78.00	935.56	904.22	923.26	933.43	965.52	973.94
79.00	935.20	902.59	922.45	932.45	964.42	973.42
80.00	934.00	900.99	919.94	930.31	963.34	972.45
81.00	933.10	899.53	920.05	929.81	963.21	971.91
82.00	932.93	897.45	918.84	928.61	963.25	971.90
83.00	929.99	895.00	917.91	925.98	962.62	971.05
84.00	930.03	893.14	914.90	925.69	961.76	970.32
85.00	929.25	891.62	914.13	925.47	960.25	970.85
86.00	926.52	889.42	911.38	924.30	959.86	971.54
87.00	926.15	888.21	908.90	923.17	958.87	971.01
88.00	926.29	886.42	906.44	922.63	957.83	969.23
89.00	924.99	885.08	904.17	921.87	956.70	969.93
90.00	922.83	884.61	901.65	921.55	956.03	968.86
91.00	922.40	883.48	898.64	920.83	954.43	968.36
92.00	919.40	882.46	894.98	920.51	954.58	968.20
93.00	919.17	881.25	892.10	919.41	952.72	967.60
94.00	918.26	880.45	890.12	918.24	951.00	967.30
95.00	916.54	879.65	887.57	918.33	951.06	966.90
96.00	915.78	879.54	885.96	917.84	950.10	967.09
97.00	915.00	878.05	883.72	917.77	949.95	965.71
98.00	914.14	876.87	882.31	917.10	949.62	966.04
99.00	913.38	876.10	880.67	915.90	948.50	964.85
100.00	912.35	875.05	879.49	915.50	948.32	965.88
100.25	912.62	874.78	879.35	915.98	948.36	965.87
100.25	912.62	874.78	879.35	915.98	948.36	965.87
101.00	911.39	873.87	877.88	915.63	947.69	964.65

102.00	910.35	873.54	876.81	914.56	947.77	964.86
103.00	909.00	873.19	874.60	914.82	946.63	963.87
104.00	908.06	871.63	873.08	913.32	946.72	964.58
105.00	907.03	870.74	871.89	912.95	945.27	964.20
106.00	904.55	869.33	869.58	912.38	945.19	963.87
107.00	904.04	867.31	868.13	911.05	943.94	964.09
108.00	902.60	865.29	867.79	910.98	942.39	963.90
109.00	901.46	863.51	866.33	910.14	942.91	963.40
110.00	900.02	861.38	864.30	908.85	941.71	963.41
111.00	898.19	860.57	863.41	907.68	941.00	962.45
112.00	897.50	859.76	861.73	906.65	941.12	962.18
113.00	897.36	858.05	861.15	904.70	940.82	961.31
114.00	896.93	856.94	859.56	903.31	939.99	961.28
115.00	894.24	854.77	858.83	902.40	939.83	960.60
116.00	893.65	853.54	857.35	901.49	939.04	960.34
117.00	891.99	851.97	856.74	900.72	936.72	959.33
118.00	889.72	850.59	854.72	899.43	936.59	957.79
119.00	889.36	849.39	854.73	897.45	935.94	957.08
120.00	886.90	847.46	854.07	896.43	934.94	956.89
121.00	885.59	847.05	853.27	894.76	934.25	954.69
122.00	884.35	846.03	852.13	893.06	934.03	954.20
123.00	881.77	845.32	850.68	891.88	934.30	952.03
124.00	880.26	844.52	851.09	890.53	931.62	952.19
125.00	878.77	843.52	849.05	888.97	931.96	951.50
126.00	876.28	842.26	848.41	888.80	932.53	949.73
127.00	874.20	841.02	847.55	887.78	930.77	949.18
128.00	871.51	840.80	845.48	885.02	930.19	948.71
129.00	870.35	839.36	844.77	883.01	929.95	947.41
130.00	868.72	837.74	843.68	881.00	930.55	945.66
131.00	866.49	836.64	842.27	879.94	929.88	945.56
132.00	864.54	836.09	842.23	879.28	929.01	944.61
133.00	863.04	834.57	840.60	876.82	929.21	944.14
134.00	860.33	833.29	839.03	876.23	928.17	942.73
135.00	857.02	832.59	836.63	875.38	927.15	940.78
136.00	856.05	830.72	836.24	873.17	926.87	939.99
137.00	854.04	830.05	834.11	872.43	925.72	938.59
138.00	853.26	829.95	832.67	871.08	924.87	938.20
139.00	850.92	828.54	832.49	868.99	924.29	936.62
140.00	849.87	828.11	831.54	868.46	923.56	935.11
141.00	847.86	826.44	830.62	866.67	923.29	933.23
142.00	847.45	826.56	829.49	866.41	922.62	931.91
143.00	845.96	826.30	828.24	864.98	920.46	931.09
144.00	844.55	824.99	828.23	864.32	920.12	929.77
145.00	841.60	825.07	826.81	862.40	919.11	928.97
146.00	840.41	824.40	826.61	862.15	918.21	927.83
147.00	839.68	823.58	825.38	860.74	916.82	926.12
148.00	838.03	823.11	824.10	859.79	915.52	925.60
149.00	836.75	822.25	824.14	859.55	913.45	924.50
150.00	835.51	821.51	823.67	858.80	913.75	922.66
151.00	832.94	821.28	823.03	857.73	913.43	922.20
152.00	831.47	820.38	821.63	856.41	911.08	920.30
153.00	830.42	821.40	820.92	855.53	910.20	918.36
154.00	828.92	820.31	820.01	854.22	909.16	916.12
155.00	827.79	819.59	819.06	853.64	907.86	915.66

156.00	826.90	818.47	817.48	851.37	906.17	913.59
157.00	825.75	818.45	816.92	851.21	905.46	912.25
158.00	825.23	818.57	816.11	848.71	903.64	910.93
159.00	824.04	818.54	814.78	847.69	903.93	908.49
160.00	822.71	817.98	814.29	847.71	902.87	906.69
161.00	821.50	818.04	813.65	846.21	900.83	904.39
162.00	821.03	816.99	813.38	845.02	900.22	902.39
163.00	819.64	816.45	812.32	843.98	897.96	899.31
164.00	818.40	815.86	812.17	842.02	897.99	897.98
165.00	818.16	815.49	811.86	842.19	897.02	894.96
166.00	817.34	815.93	811.35	839.04	894.44	892.05
167.00	817.31	815.73	810.38	837.16	892.97	888.85
168.00	816.43	815.20	810.15	835.87	890.64	887.25
169.00	816.30	815.05	809.99	834.36	889.98	883.48
170.00	815.88	814.00	809.48	833.57	887.62	880.81
171.00	815.97	814.04	809.04	832.75	886.77	877.77
172.00	816.03	814.30	808.95	831.96	884.09	874.48
173.00	816.04	814.05	808.63	830.88	883.48	872.54
174.00	814.73	813.74	807.88	829.81	881.25	871.66
175.00	814.61	812.95	807.59	829.00	880.74	868.81
176.00	815.19	813.01	807.49	828.22	879.17	866.14
177.00	814.07	812.43	807.14	826.98	878.08	863.69
178.00	814.35	811.94	807.24	826.52	876.44	861.78
179.00	813.79	811.64	806.64	825.50	873.57	859.28
180.00	813.06	811.12	806.67	824.63	871.95	858.78
181.00	813.34	810.65	806.80	824.27	871.50	856.16
182.00	813.10	809.96	805.92	824.15	870.30	854.33
183.00	812.41	809.65	805.61	823.01	868.32	852.01
184.00	811.21	809.91	805.38	822.08	867.57	850.25
185.00	811.03	808.61	805.53	820.80	865.26	848.10
186.00	811.02	808.15	805.50	819.83	864.72	844.58
187.00	810.58	807.85	804.30	818.80	863.47	842.99
188.00	809.72	808.41	804.82	818.26	862.16	841.64
189.00	810.11	807.27	804.50	817.09	861.97	839.19
190.00	809.68	807.11	804.16	817.04	860.79	837.18
191.00	810.37	806.71	803.99	816.65	859.54	834.99
192.00	809.29	806.73	803.48	816.34	858.74	833.59
193.00	809.14	806.50	803.28	815.40	858.33	831.53
194.00	809.16	806.14	803.71	814.38	856.47	831.12
195.00	808.20	806.91	803.13	813.83	855.62	828.84
196.00	808.93	805.08	802.66	812.75	855.25	828.39
197.00	808.53	805.62	802.91	811.78	855.26	827.41
198.00	808.58	805.36	802.89	810.66	854.23	825.18
199.00	808.46	805.03	802.16	810.10	853.81	823.96
200.00	808.14	804.73	802.30	809.82	852.40	822.48
201.00	808.78	805.49	802.35	809.10	851.95	821.83
202.00	808.12	804.48	802.54	808.64	849.78	821.12
203.00	807.60	804.32	801.93	808.40	848.86	820.15
204.00	807.47	804.18	801.31	808.49	846.64	819.17
205.00	807.88	803.31	801.86	807.00	846.19	817.96
206.00	807.19	804.16	800.81	806.73	844.99	816.39
207.00	807.67	803.29	801.40	805.85	845.30	815.19
208.00	806.93	803.35	801.14	805.50	843.25	814.30
209.00	807.04	802.46	800.78	804.63	842.09	812.88

210.00	806.72	802.62	800.74	803.31	840.62	812.01
211.00	806.72	802.65	800.36	803.15	840.20	812.16
212.00	807.15	801.97	800.26	801.76	838.44	812.02
213.00	806.25	801.37	799.33	801.79	837.89	811.32
214.00	805.42	801.18	799.30	801.74	835.85	810.90
215.00	806.13	801.08	798.42	801.26	835.38	809.60
216.00	806.05	800.86	798.41	801.27	833.75	808.28
217.00	806.15	800.60	798.96	801.12	833.47	808.26
218.00	805.80	800.61	798.38	800.36	832.74	808.20
219.00	805.97	800.02	798.22	799.74	831.07	807.80
220.00	804.97	799.70	797.91	798.97	830.46	807.38
221.00	805.66	799.48	797.18	797.91	828.71	807.17
222.00	804.87	799.80	796.96	797.22	828.33	806.45
223.00	805.61	799.16	797.53	796.73	826.91	805.99
224.00	805.36	798.94	796.91	795.42	825.88	805.23
225.00	804.90	798.50	796.77	795.65	826.02	804.74
226.00	804.66	798.55	796.56	794.36	825.03	804.89
227.00	804.32	798.73	795.84	793.74	824.39	804.34
228.00	804.02	797.98	795.92	793.28	823.46	804.20
229.00	803.66	798.02	795.18	792.74	823.31	803.15
230.00	804.21	797.52	795.25	792.59	822.53	802.74
231.00	803.85	797.72	795.31	791.81	822.05	801.88
232.00	803.66	797.64	795.27	791.87	821.48	802.22
233.00	803.95	798.05	794.96	791.12	820.27	801.24
234.00	804.25	797.95	794.37	790.47	819.94	800.80
235.00	803.93	797.58	793.75	790.72	819.18	799.79
236.00	803.31	797.29	794.06	789.35	819.54	799.91
237.00	803.12	797.33	793.60	789.16	817.78	799.34
238.00	803.22	796.80	793.57	788.79	818.40	799.58
239.00	803.55	796.90	793.66	788.14	817.69	798.37
240.00	803.00	796.62	792.67	787.66	817.24	797.92
241.00	803.22	796.05	792.82	788.18	816.71	798.02
242.00	802.45	796.43	792.71	787.21	817.43	797.73
243.00	802.33	795.36	792.33	786.73	817.20	797.21
244.00	802.50	795.34	792.49	786.55	815.59	796.71
245.00	802.03	795.89	792.00	787.11	814.54	796.09
246.00	801.76	796.37	791.56	786.83	815.26	795.40
247.00	801.23	795.73	791.52	786.52	815.01	794.84
248.00	801.55	795.70	792.08	786.01	814.48	794.19
249.00	801.84	795.27	791.18	785.92	813.87	794.37
250.00	801.43	795.02	790.69	785.26	813.00	794.29
251.00	800.96	795.03	790.72	784.60	812.22	794.20
252.00	800.64	794.60	790.77	784.82	812.20	793.81
253.00	800.13	794.74	790.61	784.86	811.58	793.35
254.00	799.57	794.74	790.33	784.48	811.03	792.24
255.00	799.75	795.25	790.76	784.35	810.26	792.70
256.00	799.76	795.26	790.30	784.24	810.55	791.99
257.00	799.97	794.62	790.31	784.24	810.23	792.19
258.00	799.37	794.39	790.70	783.70	810.24	791.35
259.00	798.15	793.99	790.55	783.29	808.91	791.46
260.00	798.14	793.96	789.85	783.64	808.07	791.38
261.00	797.85	794.16	790.38	783.39	807.36	790.25
262.00	797.89	794.32	790.33	783.21	806.88	789.46
263.00	797.57	793.51	789.87	782.56	805.71	789.44

264.00	797.02	793.34	789.69	782.64	805.24	788.56
265.00	797.21	793.46	789.71	782.63	804.82	788.39
266.00	796.27	793.19	789.76	782.79	803.70	788.14
267.00	795.73	793.13	789.50	782.27	803.75	786.96
268.00	795.44	793.53	788.93	782.24	803.62	786.04
269.00	795.21	793.17	789.29	782.03	803.64	786.54
270.00	795.13	793.15	788.65	781.59	802.71	785.69
271.00	795.24	792.52	788.80	781.43	802.63	785.82
272.00	794.07	792.89	788.19	781.41	801.98	786.27
273.00	793.62	791.62	788.25	781.36	801.98	785.79
274.00	793.65	791.92	788.50	781.50	802.42	785.92
275.00	793.06	792.13	788.43	780.59	802.04	785.29
276.00	793.05	791.01	787.97	780.96	801.14	784.75
277.00	792.99	791.15	788.09	779.97	800.89	784.94
278.00	793.08	790.53	787.73	780.07	800.82	784.61
279.00	792.38	791.24	787.97	779.67	800.15	784.44
280.00	792.69	790.66	787.28	779.54	798.90	784.84
281.00	792.75	790.60	787.59	779.77	799.18	785.07
282.00	792.52	791.24	787.44	779.32	798.91	784.57
283.00	792.73	790.49	787.36	779.03	799.23	783.87
284.00	791.87	790.10	788.03	779.25	797.98	784.27
285.00	792.13	789.99	787.70	779.53	798.57	783.83
286.00	792.12	790.12	787.62	779.38	798.52	783.61
287.00	791.75	790.21	787.92	778.96	798.27	783.44
288.00	791.79	789.91	788.07	778.78	798.47	783.40
289.00	791.83	790.37	788.00	778.36	797.97	782.00
290.00	791.41	790.66	787.32	778.63	797.72	782.32
291.00	790.89	790.26	787.93	778.62	796.95	782.21
292.00	790.86	789.96	787.89	778.86	797.21	782.34
293.00	790.48	789.64	787.75	778.70	796.12	781.72
294.00	790.29	789.79	786.99	778.69	795.47	781.82
295.00	790.64	790.47	787.79	778.41	794.43	781.50
296.00	790.81	789.89	787.40	778.03	794.89	781.32
297.00	790.19	788.93	787.46	778.48	794.11	780.88
298.00	790.39	789.62	787.20	778.81	793.23	780.58
299.00	790.12	788.65	787.17	778.63	792.23	780.76
300.00	790.43	789.14	786.63	778.11	791.90	780.80
301.00	790.08	789.04	787.34	778.09	791.45	780.36
302.00	789.62	788.79	787.15	778.00	791.58	779.53
303.00	789.60	788.12	787.16	778.13	790.68	779.83
304.00	789.76	788.74	787.39	778.08	791.12	779.04
305.00	790.14	788.29	787.02	777.92	790.40	778.65
306.00	790.62	788.19	787.00	777.76	789.28	779.67
307.00	790.56	787.95	787.54	777.74	789.00	779.07
308.00	789.21	787.52	787.39	777.84	788.80	779.10
309.00	789.56	787.90	787.43	778.01	788.65	778.46
310.00	789.69	787.22	787.14	778.29	788.63	778.23
311.00	789.60	788.59	787.11	777.64	787.76	778.31
312.00	790.21	787.86	786.61	777.98	787.61	777.70
313.00	789.66	787.54	786.61	777.83	787.46	777.73
314.00	789.66	787.23	786.35	778.02	787.04	777.13
315.00	788.77	787.00	786.59	777.45	786.77	777.14
316.00	789.01	786.93	786.69	777.67	786.44	776.82
317.00	788.68	787.23	786.43	777.20	786.18	776.90

318.00	789.17	787.00	786.78	777.85	785.65	777.65
319.00	789.18	786.65	786.40	777.17	785.82	777.66
320.00	788.69	786.62	786.33	777.00	785.77	777.52
321.00	788.69	786.63	786.38	777.00	785.86	776.74
322.00	788.96	786.57	785.72	776.77	785.55	776.78
323.00	788.37	785.74	785.73	776.75	785.91	776.59
324.00	788.47	786.79	785.88	777.20	785.63	776.93
325.00	788.16	785.71	786.27	777.56	785.56	776.61
326.00	788.44	786.35	785.86	777.19	785.51	777.22
327.00	788.54	785.28	785.46	777.10	785.04	777.10
328.00	788.66	785.81	785.97	776.89	785.67	777.34
329.00	789.02	784.56	786.11	776.99	785.48	777.49
330.00	789.40	784.94	785.98	777.18	785.16	777.27
331.00	788.74	784.91	785.63	777.17	785.66	776.84
332.00	788.64	785.02	785.72	777.03	785.68	776.96
333.00	788.75	784.56	785.81	777.01	785.28	777.15
334.00	789.55	785.06	785.50	777.05	784.98	777.61
335.00	788.64	785.27	785.29	777.04	785.32	777.38
336.00	789.39	784.63	785.64	777.09	784.54	777.12
337.00	788.52	784.50	785.18	777.14	784.77	777.22
338.00	788.81	784.11	786.05	777.10	784.80	777.39
339.00	788.43	784.48	785.98	776.65	784.73	777.50
340.00	788.19	784.80	785.88	777.06	784.06	777.39
341.00	788.97	783.55	785.84	776.96	783.55	776.93
342.00	788.55	784.38	785.26	776.69	783.46	777.25
343.00	788.99	783.69	785.75	776.95	783.33	777.23
344.00	788.55	783.62	787.01	777.51	782.82	777.19
345.00	788.67	784.14	785.76	776.89	783.03	778.00
346.00	788.52	783.83	785.76	777.35	782.97	777.46
347.00	788.93	784.21	786.13	777.24	782.64	777.28
348.00	788.99	784.15	785.30	777.02	782.20	776.37
349.00	789.01	784.63	785.97	777.22	782.37	776.39
350.00	788.70	783.70	786.07	776.67	781.68	776.45
351.00	788.46	784.01	786.53	776.92	782.07	777.33
352.00	787.92	782.99	786.39	777.21	782.04	776.96
353.00	788.71	783.72	786.05	777.34	782.29	777.00
354.00	789.56	783.36	785.84	776.74	782.56	776.89
355.00	788.92	783.48	786.36	777.10	781.96	776.89
356.00	788.84	783.56	786.56	776.58	781.55	777.44
357.00	788.67	784.07	786.14	777.43	781.85	777.04
358.00	788.45	783.97	785.90	777.20	781.63	777.34
359.00	788.32	784.47	786.44	777.25	781.63	777.58
360.00	788.68	783.68	786.41	777.24	781.66	777.12
361.00	788.17	784.33	785.90	777.44	781.77	777.63
362.00	788.23	783.81	786.66	777.98	780.91	777.51
363.00	787.64	783.12	786.64	777.97	781.04	777.48
364.00	787.84	783.32	786.98	777.91	780.32	777.55
365.00	787.52	783.26	787.03	778.01	780.98	777.23
366.00	786.86	783.71	786.74	778.00	780.97	777.08
367.00	787.13	783.32	786.63	777.73	780.78	777.49
368.00	786.43	783.23	787.21	777.79	780.34	777.41
369.00	787.19	783.43	787.45	777.84	780.38	776.84
370.00	787.31	783.12	786.05	778.27	780.47	777.43
371.00	787.62	782.92	786.91	777.97	780.13	777.78

372.00	787.18	783.45	787.14	777.24	780.14	777.56
373.00	786.54	783.11	787.37	777.71	779.89	777.59
374.00	786.32	783.06	787.38	778.11	780.03	776.73
375.00	786.28	782.95	787.52	777.85	780.05	777.22
376.00	787.10	783.16	787.65	777.49	779.97	777.49
377.00	786.38	783.05	787.51	778.14	779.77	777.72
378.00	786.61	782.91	787.42	778.50	779.82	777.55
379.00	785.92	783.37	788.18	778.68	780.11	777.49
380.00	786.30	782.62	787.66	778.79	779.79	777.80
381.00	786.71	783.12	787.45	778.59	779.83	777.88
382.00	786.58	783.09	786.80	778.14	780.21	777.81
383.00	786.65	783.13	786.66	778.52	779.97	776.92
384.00	785.80	783.35	788.02	779.18	780.05	777.55
385.00	786.37	782.78	787.12	778.49	779.18	777.58
386.00	786.29	783.24	787.17	779.00	779.45	777.62
387.00	786.04	783.07	788.14	778.64	780.11	777.06
388.00	787.12	783.63	788.00	778.48	779.41	777.08
389.00	786.52	783.44	787.44	778.65	779.56	777.71
390.00	785.76	783.18	788.07	778.64	779.79	777.74
391.00	786.46	783.00	787.89	779.27	779.75	777.94
392.00	785.74	783.20	788.10	779.40	779.92	777.31
393.00	786.08	782.36	788.24	779.18	779.61	778.19
394.00	786.74	782.53	788.02	779.53	780.01	777.18
395.00	786.53	782.59	788.32	779.74	779.44	777.43
396.00	786.17	783.30	788.37	779.62	779.06	776.65
397.00	786.59	783.19	787.80	780.01	778.76	777.33
398.00	787.15	782.76	788.76	779.60	779.08	776.83
399.00	786.66	783.00	788.23	779.66	778.84	777.29
400.00	786.92	782.88	788.37	779.87	779.09	776.98
400.25	786.89	782.75	789.18	779.32	779.55	777.66
400.25	786.89	782.75	789.18	779.32	779.55	777.66
401.00	786.17	782.86	789.12	779.68	779.19	777.64
402.00	786.12	782.52	789.08	779.66	779.59	777.56
403.00	786.64	781.84	788.60	779.91	778.97	777.67
404.00	786.97	782.16	788.42	778.98	778.42	777.05
405.00	786.66	782.30	788.87	779.53	778.16	776.70
406.00	786.39	782.55	788.40	779.05	777.97	776.19
407.00	786.12	782.17	788.67	778.90	777.07	776.87
408.00	786.92	782.49	788.13	778.02	777.84	776.75
409.00	786.32	782.13	787.97	778.54	777.86	776.98
410.00	786.45	782.28	787.85	779.12	777.10	777.06
411.00	785.64	782.60	788.02	778.84	776.85	777.04
412.00	785.51	782.33	787.36	779.12	777.20	777.35
413.00	785.61	782.10	787.24	779.22	777.34	777.15
414.00	785.93	782.17	787.99	779.61	776.83	777.55
415.00	786.56	782.63	788.05	779.07	776.84	777.21
416.00	785.80	782.63	788.09	779.03	777.29	777.06
417.00	786.48	782.28	788.23	779.12	776.79	777.25
418.00	785.59	781.64	788.02	778.63	776.76	777.20
419.00	785.46	781.61	787.57	779.08	776.51	777.04
420.00	785.80	781.98	787.92	779.17	775.84	777.11
421.00	785.93	782.38	788.42	778.76	776.07	776.80
422.00	785.18	781.63	787.71	779.03	776.31	776.87
423.00	785.54	781.72	787.49	779.18	776.14	777.31

424.00	785.56	782.11	787.65	778.88	776.34	777.46
425.00	785.45	782.08	787.44	778.80	775.63	777.36
426.00	785.12	782.40	787.31	778.49	775.47	776.87
427.00	785.09	782.32	787.70	778.99	775.70	777.01
428.00	785.47	781.54	787.86	778.84	774.98	776.80
429.00	785.32	781.43	787.84	778.89	775.09	777.18
430.00	785.47	781.50	788.46	777.90	774.94	776.89
431.00	785.42	781.38	787.66	777.93	775.19	776.48
432.00	784.56	781.45	788.19	778.70	775.08	776.67
433.00	784.78	782.03	788.02	778.60	775.04	777.03
434.00	784.51	782.09	788.18	778.78	775.20	777.32
435.00	784.60	781.91	788.24	778.73	774.98	777.00
436.00	784.81	781.89	787.58	778.62	775.54	776.80
437.00	784.93	781.42	787.77	778.33	774.59	776.37
438.00	784.58	781.26	787.61	778.23	774.88	776.14
439.00	784.18	781.52	786.99	778.90	774.55	776.31
440.00	784.16	780.63	787.56	778.36	774.35	776.44
441.00	784.30	780.77	786.95	778.66	774.53	777.17
442.00	783.93	781.23	787.30	778.12	775.27	776.91
443.00	784.17	781.22	787.85	778.25	775.97	777.00
444.00	784.34	781.40	787.88	778.02	775.10	777.44
445.00	784.40	781.47	788.20	778.33	774.83	776.47
446.00	784.51	781.48	787.23	778.89	774.91	776.63
447.00	784.01	782.08	787.72	778.95	774.16	776.77
448.00	783.77	781.77	787.75	778.95	773.70	776.81
449.00	783.74	781.46	787.67	778.29	774.36	776.62
450.00	784.09	781.29	787.39	778.65	774.88	777.42
451.00	784.29	780.91	787.52	778.69	774.85	777.14
452.00	784.08	781.28	786.73	778.76	774.61	776.86
453.00	784.61	781.37	787.07	778.68	774.31	776.36
454.00	783.78	781.44	787.40	778.35	774.71	776.38
455.00	782.91	781.48	787.63	778.14	774.93	776.75
456.00	783.76	781.19	787.99	778.51	774.94	776.18
457.00	784.27	781.08	787.79	778.73	774.60	776.69
458.00	784.23	780.70	787.87	778.22	774.89	777.33
459.00	783.89	780.52	787.98	778.16	774.70	776.78
460.00	784.05	780.81	787.50	778.83	774.30	776.83
461.00	784.51	781.22	788.01	778.34	774.31	776.83
462.00	784.15	781.02	787.36	778.49	774.64	776.74
463.00	783.91	781.79	787.45	778.69	774.65	776.59
464.00	784.01	781.06	787.09	778.70	774.67	776.70
465.00	783.61	781.14	787.11	778.12	774.22	776.38
466.00	783.49	781.44	787.14	778.80	774.56	776.27
467.00	784.11	780.79	786.89	778.12	774.48	776.10
468.00	784.26	781.09	786.75	777.90	774.50	776.63
469.00	783.97	780.36	786.89	778.24	774.48	775.95
470.00	784.05	780.41	786.70	778.35	774.21	776.89
471.00	783.93	780.45	787.09	778.52	774.31	776.45
472.00	783.56	781.14	787.53	777.70	773.86	776.11
473.00	783.21	780.63	786.96	778.86	774.61	776.19
474.00	783.45	780.97	787.24	777.80	774.92	776.38
475.00	783.61	780.31	787.19	778.73	774.79	776.59
476.00	783.97	781.05	787.23	778.76	774.79	776.26
477.00	783.75	780.93	786.74	778.65	774.37	776.13

478.00	783.84	780.95	787.11	778.42	775.04	776.17
479.00	783.20	780.87	787.28	778.06	774.65	776.15
480.00	783.71	780.45	787.70	777.98	774.56	776.66
481.00	783.58	781.13	787.17	778.25	774.48	776.39
482.00	783.15	780.99	787.21	778.27	774.26	776.73
483.00	783.29	780.76	787.11	778.80	774.44	776.41
484.00	783.61	780.49	787.50	778.16	773.93	776.71
485.00	783.34	780.36	787.66	778.64	774.34	776.48
486.00	783.66	779.98	787.18	777.75	774.52	775.82
487.00	783.30	780.05	786.73	778.37	774.81	776.58
488.00	783.23	780.61	786.52	778.20	774.85	776.52
489.00	783.82	780.24	787.31	778.46	774.62	776.52
490.00	783.71	780.46	787.19	777.81	774.23	776.45
491.00	783.94	780.73	787.56	778.52	774.05	776.42
492.00	783.83	780.53	786.87	777.85	774.20	776.55
493.00	784.04	780.88	787.82	778.00	773.56	776.39
494.00	783.64	780.08	786.79	778.48	774.35	776.70
495.00	783.04	780.46	787.46	777.56	774.34	776.30
496.00	783.04	780.62	787.25	778.19	774.26	776.80
497.00	783.84	780.35	787.35	778.18	774.45	776.99
498.00	783.19	780.91	787.30	778.80	774.29	776.39
499.00	782.99	780.66	786.42	778.22	774.44	776.52
500.00	783.64	780.01	786.97	778.45	773.62	776.07
501.00	783.76	780.11	787.38	778.63	774.80	776.22
502.00	783.72	780.16	787.34	777.92	774.46	775.89
503.00	783.54	779.71	786.72	778.42	774.10	776.03
504.00	783.00	780.51	786.15	778.46	774.02	775.87
505.00	783.13	780.57	786.95	778.12	774.23	776.24
506.00	782.79	779.79	786.66	778.05	774.51	776.25
507.00	783.47	780.24	786.42	778.03	774.21	776.33
508.00	784.05	780.14	786.63	778.05	774.43	776.11
509.00	783.96	780.11	786.28	778.43	774.64	776.42
510.00	783.86	780.26	786.76	778.57	774.57	776.42
511.00	782.97	779.90	786.42	778.73	775.06	776.65
512.00	783.02	779.73	787.14	778.17	774.34	776.98
513.00	782.95	780.26	787.09	777.89	773.49	777.14
514.00	783.37	780.44	787.23	778.03	774.16	776.23
515.00	783.54	780.44	786.46	777.55	773.94	776.09
516.00	784.50	780.54	786.86	778.20	774.15	775.97
517.00	784.31	779.75	786.69	777.62	774.29	775.86
518.00	783.24	780.22	786.15	777.73	774.21	776.46
519.00	783.62	780.29	786.07	778.00	773.92	775.80
520.00	783.45	779.99	786.59	777.62	774.31	776.22
521.00	783.14	779.99	786.57	778.17	774.44	776.39
522.00	783.39	779.48	786.47	777.67	774.01	775.57
523.00	782.89	779.53	786.49	778.06	774.83	776.17
524.00	783.12	780.49	786.41	777.92	774.28	776.63
525.00	783.02	780.53	786.54	777.16	773.78	776.05
526.00	783.23	779.62	785.87	777.95	773.97	776.40
527.00	783.10	779.85	786.73	778.42	774.47	776.74
528.00	783.73	779.95	786.90	778.19	774.70	776.05
529.00	783.69	779.98	786.69	778.62	774.14	776.00
530.00	784.00	779.55	786.22	777.59	774.72	775.99
531.00	783.28	780.17	786.17	778.03	774.24	776.57

532.00	783.16	780.06	786.42	778.44	774.40	776.65
533.00	783.12	780.21	786.00	778.34	774.67	776.53
534.00	783.90	780.26	786.09	778.19	774.46	776.34
535.00	783.52	779.97	786.24	778.31	774.61	775.87
536.00	783.51	780.26	786.19	778.79	773.87	776.44
537.00	783.12	780.21	785.78	778.36	774.04	776.40
538.00	783.62	779.98	786.55	778.11	774.17	776.16
539.00	783.25	780.18	786.57	777.85	773.95	775.97
540.00	783.73	780.19	786.55	777.91	774.66	776.68
541.00	783.32	779.64	786.19	777.70	774.06	775.98
542.00	783.55	780.39	786.13	777.91	773.80	776.24
543.00	783.19	780.46	785.71	777.43	774.13	776.20
544.00	783.42	779.95	785.46	777.46	774.01	775.78
545.00	783.19	779.82	785.67	777.39	774.29	775.98
546.00	784.02	779.59	786.29	777.87	773.66	775.72
547.00	783.57	779.64	786.00	777.91	774.48	776.24
548.00	783.55	779.48	785.57	778.37	774.16	775.78
549.00	783.43	779.86	785.91	777.97	774.13	775.81
550.00	783.03	779.68	785.96	778.77	773.89	776.07
551.00	783.19	779.66	785.67	778.19	774.37	776.19
552.00	782.89	779.10	786.10	777.43	774.13	776.24
553.00	783.79	779.27	785.82	778.49	773.80	776.85
554.00	783.80	779.38	784.91	778.68	774.47	776.62
555.00	783.57	779.81	785.43	778.23	774.04	776.00
556.00	783.57	779.53	785.82	777.61	773.58	775.88
557.00	783.16	779.64	785.84	777.94	773.07	775.96
558.00	783.25	779.85	785.59	777.62	773.78	776.28
559.00	783.58	779.90	785.87	778.05	774.11	776.63
560.00	783.35	779.83	785.95	778.33	773.92	776.62
561.00	783.52	779.40	785.49	777.90	773.90	776.36
562.00	784.31	779.90	785.73	777.80	774.31	776.38
563.00	783.69	779.87	786.02	777.97	773.62	776.13
564.00	783.02	780.32	785.57	777.80	773.71	776.09
565.00	783.29	779.77	785.28	777.90	774.56	775.88
566.00	783.46	779.40	785.01	777.14	774.28	775.99
567.00	783.90	779.72	786.18	777.42	773.97	776.34
568.00	783.44	779.52	785.83	777.52	774.32	775.78
569.00	783.62	779.31	785.24	778.26	774.43	775.75
570.00	783.12	780.08	785.79	778.01	773.84	775.84
571.00	782.89	780.15	785.54	777.38	774.38	775.64
572.00	782.82	780.01	786.15	777.21	774.11	775.51
573.00	783.29	779.34	785.92	777.18	774.39	775.75
574.00	783.62	779.58	785.62	777.33	773.40	775.75
575.00	783.38	779.75	785.31	777.96	773.68	775.90
576.00	783.02	779.32	785.23	777.81	773.32	776.37
577.00	782.87	779.06	785.62	777.98	774.02	776.30
578.00	783.45	779.22	785.82	777.82	773.90	776.14
579.00	783.12	779.40	785.55	778.05	773.88	775.85
580.00	783.28	779.73	785.88	777.81	773.65	776.15
581.00	783.44	779.39	786.06	777.87	773.53	775.96
582.00	783.14	779.52	785.86	777.48	773.68	775.96
583.00	783.34	779.06	785.72	778.04	774.05	776.07
584.00	783.57	779.81	785.62	778.11	774.36	775.17
585.00	783.22	779.11	785.87	777.89	774.58	775.29

586.00	783.49	779.53	785.59	778.09	773.70	776.33
587.00	783.48	779.56	785.44	778.04	774.53	776.26
588.00	783.65	779.50	785.40	778.01	773.87	775.98
589.00	783.15	779.21	785.83	777.74	772.99	776.02
590.00	782.69	779.22	785.57	777.88	774.03	775.30
591.00	782.96	779.15	785.68	777.71	774.15	775.97
592.00	783.04	779.66	785.69	778.16	773.87	776.28
593.00	782.72	779.93	785.78	777.67	773.96	776.00
594.00	782.58	779.84	785.63	777.53	773.46	775.69
595.00	783.03	780.03	785.75	778.35	774.00	775.63
596.00	783.29	779.57	785.49	777.92	773.77	776.22
597.00	783.69	780.28	785.44	777.88	773.69	775.79
598.00	783.14	779.99	785.62	777.37	773.73	775.74
599.00	783.77	779.54	785.62	777.16	774.60	775.73
600.00	783.98	779.02	785.77	777.47	774.85	775.67
601.00	783.15	779.31	785.16	777.73	774.15	775.47
602.00	783.84	779.29	785.68	777.75	773.85	775.05
603.00	783.51	779.80	785.16	777.79	774.73	775.54
604.00	783.73	780.21	785.38	777.94	773.68	776.10
605.00	783.34	779.88	785.50	778.08	773.78	776.12
606.00	783.53	779.66	785.32	777.81	774.15	776.34
607.00	783.06	779.86	785.31	777.78	774.50	776.13
608.00	782.96	779.48	785.92	777.42	774.41	775.59
609.00	783.07	779.06	785.57	777.94	774.07	775.67
610.00	783.36	779.00	785.87	777.47	773.89	775.61
611.00	783.33	778.89	785.67	777.64	774.69	775.82
612.00	783.26	779.29	785.63	777.44	774.29	775.41
613.00	783.42	779.61	785.78	777.47	773.67	775.71
614.00	783.46	779.24	785.19	777.25	773.66	775.79
615.00	783.28	778.86	785.16	777.45	773.36	775.89
616.00	782.94	778.88	784.85	777.51	773.86	776.46
617.00	782.98	779.24	785.12	777.75	774.45	776.15
618.00	782.72	779.00	785.11	778.30	774.29	775.92
619.00	783.35	779.60	785.63	778.03	773.58	776.03
620.00	783.28	779.59	785.00	777.38	773.60	776.03
621.00	783.00	779.26	785.57	777.12	773.59	776.31
622.00	783.50	778.98	785.49	777.54	773.46	775.88
623.00	783.58	779.27	785.75	777.25	774.26	775.66
624.00	783.39	779.25	785.54	777.72	774.11	776.09
625.00	783.08	779.03	785.00	777.65	774.82	775.84
626.00	783.03	778.97	785.06	777.14	774.28	776.02
627.00	782.66	779.21	785.20	777.10	773.74	775.90
628.00	782.85	779.20	785.24	777.47	774.21	775.63
629.00	783.02	779.90	785.03	777.22	774.02	776.35
630.00	783.02	779.49	785.39	777.50	774.17	775.91
631.00	783.02	779.57	785.20	777.48	773.87	776.40
632.00	782.96	779.44	785.40	778.02	773.80	776.06
633.00	783.10	778.64	785.63	777.61	774.07	775.61
634.00	783.10	779.36	785.74	777.90	773.72	775.62
635.00	783.31	779.43	785.52	777.17	773.51	775.28
636.00	782.54	779.26	785.04	777.71	773.16	775.66
637.00	783.00	778.77	785.26	777.66	773.66	775.71
638.00	783.51	778.81	784.87	777.34	773.88	775.96
639.00	783.03	779.36	785.03	777.07	774.17	775.63

640.00	783.50	779.38	785.56	777.69	773.91	775.36
641.00	783.88	779.67	785.77	777.67	774.00	775.44
642.00	783.41	779.71	785.44	777.89	773.52	776.15
643.00	783.31	780.13	785.37	777.49	773.57	776.49
644.00	783.05	779.79	784.67	777.47	773.54	775.87
645.00	782.88	779.22	784.88	777.83	773.93	776.07
646.00	783.04	779.76	784.82	777.67	773.88	775.36
647.00	782.60	779.69	784.65	777.49	773.92	775.57
648.00	782.83	779.80	785.41	777.11	774.34	775.40
649.00	782.93	779.89	784.42	776.94	774.53	775.51
650.00	782.90	779.59	784.42	777.55	774.26	775.75
651.00	782.63	779.82	784.44	777.72	774.04	775.73
652.00	783.34	779.41	784.59	777.69	773.71	775.54
653.00	783.50	779.67	784.93	778.15	774.21	775.32
654.00	783.33	779.10	784.66	777.40	774.25	775.37
655.00	783.27	779.01	785.28	777.38	773.23	775.75
656.00	783.35	778.82	785.31	777.19	774.05	775.24
657.00	783.10	779.08	784.83	777.05	774.27	775.23
658.00	782.92	779.00	785.39	777.54	774.06	775.47
659.00	782.73	779.36	784.94	777.16	773.73	775.46
660.00	783.37	779.38	784.80	777.31	774.04	776.00
661.00	783.07	779.00	784.88	777.51	774.56	775.98
662.00	782.98	779.05	784.97	777.50	774.12	775.55
663.00	783.24	779.57	784.39	777.81	773.70	775.50
664.00	783.56	779.10	784.82	778.32	773.43	775.47
665.00	783.27	779.09	784.80	777.42	773.54	776.07
666.00	782.99	779.45	784.24	777.14	773.91	775.22
667.00	782.63	779.61	784.61	777.31	773.35	775.71
668.00	781.82	779.53	784.54	777.69	773.35	776.33
669.00	782.51	779.10	785.34	778.00	773.29	775.58
670.00	783.00	779.45	784.81	777.55	773.74	775.68
671.00	782.99	779.33	785.14	777.24	773.74	775.69
672.00	783.71	779.01	784.71	776.91	774.27	775.41
673.00	783.97	779.41	784.19	777.28	774.52	775.75
674.00	782.80	779.42	784.57	777.75	774.59	775.44
675.00	783.02	779.49	784.93	777.81	774.51	775.82
676.00	783.07	780.14	784.98	778.38	773.62	775.40
677.00	782.94	779.83	784.96	777.68	773.60	775.52
678.00	783.17	779.73	784.62	777.43	773.89	775.73
679.00	782.52	779.39	784.47	778.12	773.70	775.59
680.00	783.14	779.50	785.16	778.06	774.07	775.59
681.00	782.60	779.76	784.92	778.05	774.28	775.18
682.00	783.31	778.94	784.88	777.81	774.14	775.75
683.00	783.09	778.88	784.37	777.14	773.59	776.03
684.00	782.84	779.36	784.73	777.50	773.83	775.66
685.00	783.10	779.17	784.68	776.93	774.05	775.75
686.00	782.54	779.81	785.18	776.79	774.45	775.62
687.00	782.45	779.60	784.86	776.92	774.91	775.22
688.00	782.74	779.52	785.24	777.53	774.12	774.87
689.00	782.93	779.75	784.73	777.89	773.66	775.66
690.00	783.50	779.27	784.66	777.83	773.52	775.93
691.00	783.30	779.56	784.03	777.10	774.16	775.61
692.00	783.05	779.34	784.70	776.89	773.99	775.28
693.00	783.18	779.14	784.67	776.31	773.82	775.48

694.00	783.00	779.69	784.57	777.36	773.71	775.59
695.00	783.63	779.24	785.09	777.29	773.83	775.60
696.00	783.68	779.36	784.80	777.33	773.77	774.72
697.00	783.45	779.11	784.71	777.47	773.66	774.93
698.00	783.48	778.35	784.28	776.89	773.69	775.41
699.00	783.00	779.15	784.20	777.01	773.84	775.27
700.00	782.96	778.81	784.74	777.05	773.61	775.42
700.25	783.24	779.26	785.00	776.96	773.52	775.98
700.25	783.24	779.26	785.00	776.96	773.52	775.98
701.00	783.39	779.68	784.36	777.27	773.91	775.91
702.00	783.14	779.10	784.17	777.30	774.03	775.95
703.00	783.29	778.67	784.42	777.62	773.72	775.48
704.00	782.80	779.02	784.64	777.48	772.87	775.02
705.00	782.96	778.77	784.18	776.79	773.27	774.52
706.00	782.25	778.39	784.27	776.79	773.08	774.65
707.00	782.91	778.37	784.50	775.97	773.02	774.04
708.00	782.67	778.69	783.76	776.38	773.29	774.38
709.00	782.39	778.53	783.80	776.46	773.35	774.53
710.00	782.30	778.06	783.20	776.86	772.88	774.05
711.00	782.77	778.70	782.83	776.28	772.33	774.47
712.00	781.78	778.17	782.58	775.71	772.79	774.44
713.00	781.51	778.08	782.89	775.38	772.71	774.74
714.00	781.90	777.94	782.70	775.76	773.38	774.39
715.00	781.93	777.74	782.24	775.75	772.42	773.95
716.00	782.31	776.78	782.60	775.84	772.70	773.81
717.00	781.61	777.25	782.79	776.15	772.07	773.57
718.00	781.10	777.84	782.62	776.18	772.50	773.08
719.00	780.88	777.61	782.38	775.93	772.01	773.31
720.00	780.97	777.92	782.25	775.28	773.00	773.39
721.00	780.98	777.16	782.21	775.48	772.11	773.28
722.00	781.61	777.66	781.78	775.95	771.09	773.21
723.00	781.65	777.05	782.12	775.56	771.72	773.40
724.00	780.84	776.86	782.39	774.92	771.79	773.45
725.00	780.44	776.70	781.76	774.77	771.89	773.00
726.00	780.78	776.44	782.09	774.68	771.30	772.77
727.00	780.72	776.59	782.53	774.51	771.27	772.25
728.00	780.27	775.92	781.67	774.60	771.27	772.49
729.00	780.35	776.28	781.63	774.08	771.41	772.58
730.00	780.10	776.08	781.38	773.90	771.17	772.53
731.00	780.03	776.58	781.06	774.42	770.88	772.15
732.00	780.07	776.21	781.46	773.81	770.57	772.06
733.00	780.35	776.21	781.32	774.83	770.70	772.17
734.00	779.86	775.50	781.50	774.31	770.10	772.07
735.00	779.84	776.04	781.16	774.06	770.84	772.29
736.00	779.63	776.03	781.10	774.74	771.05	771.55
737.00	779.17	775.48	780.58	773.79	770.64	772.01
738.00	779.53	775.60	780.47	773.45	770.72	772.07
739.00	779.52	775.40	780.52	773.58	770.31	771.49
740.00	779.27	776.70	780.40	772.88	770.18	771.71
741.00	778.94	775.84	780.44	772.92	769.53	771.02
742.00	778.59	775.68	780.99	773.07	769.80	770.70
743.00	778.24	775.15	780.07	773.04	770.21	770.92
744.00	778.12	775.04	780.39	773.11	770.12	770.66
745.00	778.47	775.09	780.33	773.84	769.56	770.47

746.00	778.76	775.02	780.36	773.35	769.65	770.58
747.00	778.21	774.33	780.29	772.74	769.25	770.73
748.00	778.28	774.68	779.75	772.33	769.14	770.72
749.00	778.04	774.70	779.85	772.32	769.91	770.53
750.00	778.69	774.94	780.21	772.11	769.84	770.69
751.00	778.23	774.69	779.71	772.66	769.02	770.45
752.00	778.34	774.72	779.75	772.56	769.32	770.01
753.00	778.50	774.93	779.94	772.86	768.65	770.11
754.00	777.98	774.23	779.43	772.23	768.88	769.48
755.00	777.45	774.41	779.72	771.99	769.19	769.52
756.00	777.65	774.55	779.14	771.61	769.12	769.67
757.00	777.41	774.11	778.93	771.13	768.94	769.87
758.00	777.40	773.69	778.91	771.42	768.90	769.63
759.00	777.50	773.60	779.09	771.54	768.78	769.40
760.00	777.66	774.24	779.26	771.43	768.94	768.77
761.00	777.00	773.77	779.06	771.52	768.72	769.12
762.00	776.42	773.84	778.72	771.69	767.89	768.68
763.00	776.85	773.81	778.70	771.77	768.15	768.81
764.00	776.31	773.68	778.66	771.02	768.66	768.66
765.00	776.65	773.88	778.06	770.26	768.17	768.22
766.00	776.29	773.63	778.47	770.86	768.28	768.59
767.00	776.09	773.32	778.32	770.49	767.66	768.58
768.00	776.79	773.47	778.17	770.62	767.28	768.72
769.00	776.71	772.43	778.47	770.44	767.52	768.46
770.00	776.33	772.62	778.28	770.99	767.83	768.12
771.00	775.99	772.40	777.88	770.46	767.85	768.38
772.00	775.91	772.08	777.68	770.21	768.04	767.86
773.00	775.97	772.64	777.74	770.08	767.20	768.00
774.00	775.57	772.49	777.35	769.97	767.39	767.62
775.00	775.89	772.62	777.61	770.09	767.44	767.99
776.00	775.86	772.13	777.17	769.97	767.10	767.43
777.00	775.59	772.42	776.94	769.94	767.56	767.56
778.00	775.50	772.64	776.93	769.32	767.12	767.26
779.00	775.58	772.44	777.12	769.42	766.84	767.01
780.00	775.39	771.90	776.49	769.51	766.73	767.18
781.00	775.82	772.31	776.60	769.70	766.82	767.22
782.00	775.86	771.96	776.67	769.60	766.52	767.36
783.00	775.61	771.83	776.91	769.20	766.61	766.77
784.00	775.31	771.98	776.39	769.02	766.18	766.56
785.00	775.35	771.65	776.53	768.99	766.19	766.82
786.00	775.30	771.85	776.28	769.40	766.11	766.76
787.00	775.47	771.97	775.92	768.94	766.15	766.75
788.00	774.45	771.09	776.47	768.91	765.72	766.44
789.00	774.64	771.77	775.96	768.65	765.95	766.82
790.00	774.77	771.30	776.64	768.34	765.49	766.72
791.00	774.19	771.46	776.19	768.31	765.36	766.54
792.00	774.58	770.93	776.24	768.53	765.37	766.47
793.00	774.36	771.13	776.22	768.52	765.81	766.01
794.00	774.11	771.11	776.17	768.37	765.88	766.40
795.00	773.94	770.88	775.32	768.26	765.11	765.92
796.00	773.96	770.26	775.77	768.17	765.24	765.67
797.00	773.88	770.48	775.16	768.06	765.20	765.74
798.00	773.68	770.66	775.11	768.03	765.07	765.42
799.00	774.31	770.99	774.87	767.62	765.45	765.66

800.00	773.97	770.44	775.18	767.48	765.73	765.76
801.00	774.13	769.97	774.86	767.42	764.83	765.39
802.00	773.38	769.88	775.36	767.49	764.66	765.17
803.00	773.87	769.68	775.27	767.25	764.54	764.93
804.00	773.37	770.19	775.29	767.30	764.57	765.64
805.00	773.01	769.87	775.00	767.89	764.82	764.87
806.00	772.93	769.82	774.61	767.56	764.74	765.17
807.00	773.47	769.64	774.22	767.66	764.36	764.43
808.00	773.32	769.56	774.99	766.92	764.46	764.82
809.00	772.72	769.47	774.69	767.30	764.06	764.20
810.00	772.66	769.43	774.88	767.02	764.17	764.19
811.00	772.63	769.41	774.12	766.81	763.83	764.54
812.00	772.96	769.62	774.31	766.61	763.82	764.94
813.00	772.75	768.94	774.52	766.38	763.79	764.59
814.00	772.61	769.19	773.98	766.33	764.03	764.06
815.00	772.59	768.98	773.89	766.31	763.94	763.97
816.00	772.75	768.96	773.99	766.53	763.71	764.11
817.00	772.38	768.90	773.86	765.88	763.34	764.19
818.00	771.90	769.17	773.74	765.73	762.81	763.43
819.00	772.12	768.83	773.52	766.32	762.96	763.62
820.00	771.91	768.74	773.61	766.25	763.61	763.73
821.00	772.01	768.56	773.57	766.02	763.55	763.65
822.00	772.21	768.19	773.52	765.86	763.36	763.62
823.00	772.05	768.56	773.42	766.06	763.21	763.68
824.00	771.93	768.41	773.48	765.88	762.66	763.35
825.00	771.24	768.37	773.34	765.39	762.73	763.19
826.00	771.28	768.12	773.29	765.37	762.71	762.84
827.00	771.08	768.40	772.94	764.76	762.57	763.10
828.00	770.96	768.51	773.18	765.11	762.28	763.05
829.00	771.06	768.23	772.77	765.19	762.90	762.96
830.00	771.26	767.98	772.66	765.59	762.74	763.07
831.00	771.05	768.05	772.06	765.61	762.27	762.63
832.00	770.73	767.58	772.12	764.82	762.09	762.73
833.00	770.06	767.61	772.33	764.27	762.17	762.17
834.00	770.80	767.09	772.45	764.95	762.50	762.10
835.00	770.61	767.21	772.49	764.73	762.14	761.69
836.00	770.61	766.94	772.34	765.14	762.07	762.25
837.00	770.31	766.75	772.25	764.45	761.79	762.38
838.00	770.58	766.78	772.08	764.25	761.82	761.78
839.00	770.94	766.86	772.12	764.44	761.62	761.98
840.00	770.63	767.50	772.35	764.15	761.59	762.29
841.00	770.34	766.79	771.53	763.92	761.86	762.17
842.00	770.18	766.44	771.21	763.80	761.96	761.53
843.00	769.84	766.33	771.16	763.64	761.60	761.43
844.00	770.07	766.72	770.95	763.24	761.15	761.56
845.00	770.26	766.42	771.04	763.66	760.80	761.76
846.00	769.99	766.37	770.85	763.46	761.55	761.76
847.00	769.90	766.38	771.09	763.74	761.54	761.43
848.00	769.66	766.10	770.82	763.53	760.98	761.52
849.00	769.40	766.05	771.26	763.99	760.55	761.46
850.00	769.48	766.34	771.09	763.69	761.21	761.36
851.00	769.43	766.18	771.43	763.58	760.92	760.83
852.00	769.60	765.89	770.92	762.94	760.92	761.14
853.00	769.30	765.79	770.88	763.06	760.39	760.56

854.00	769.25	766.02	770.72	763.25	760.59	760.75
855.00	769.02	766.12	770.94	763.39	760.34	760.95
856.00	769.05	766.30	770.87	762.44	760.09	761.16
857.00	768.68	765.79	770.72	762.69	760.15	760.70
858.00	768.91	765.14	770.25	762.61	759.99	760.19
859.00	769.10	765.03	770.03	762.38	760.09	760.24
860.00	768.65	765.26	770.21	762.42	760.16	760.46
861.00	768.43	765.72	769.75	762.34	760.69	760.26
862.00	768.48	765.66	769.98	762.28	759.98	759.94
863.00	768.06	765.19	770.31	762.23	759.42	759.53
864.00	767.89	765.29	769.67	762.01	759.36	759.96
865.00	767.79	764.97	769.52	762.01	759.63	759.90
866.00	767.51	764.99	769.29	761.89	759.61	760.07
867.00	767.95	764.64	769.01	761.97	759.00	759.99
868.00	767.47	764.41	768.73	762.13	759.14	759.89
869.00	767.72	764.39	769.51	761.89	759.34	759.29
870.00	767.56	764.56	769.32	761.47	759.23	759.39
871.00	767.53	764.51	769.05	761.56	759.23	759.40
872.00	767.22	764.88	768.90	761.18	759.35	759.83
873.00	767.26	764.49	768.99	761.33	758.86	759.20
874.00	767.05	764.86	768.67	761.66	759.07	759.36
875.00	767.46	764.14	769.07	761.34	759.46	759.29
876.00	767.24	764.38	768.80	760.91	758.99	758.79
877.00	766.58	763.76	768.83	760.81	758.39	758.69
878.00	767.19	763.85	769.04	760.84	758.24	758.74
879.00	766.87	763.84	768.90	761.05	758.43	758.40
880.00	766.38	763.78	768.74	761.26	758.27	758.14
881.00	766.60	763.74	768.81	760.77	758.26	758.37
882.00	766.80	763.80	768.72	760.34	758.08	758.61
883.00	766.98	763.93	768.88	759.90	758.35	758.72
884.00	766.65	763.65	768.39	760.07	758.20	758.61
885.00	766.71	763.53	768.45	760.24	758.28	758.12
886.00	766.57	763.57	768.03	760.40	757.96	758.14
887.00	766.50	763.55	767.74	760.56	758.23	758.20
888.00	766.47	762.80	767.52	760.40	757.81	757.60
889.00	766.63	763.16	767.59	759.89	758.00	757.44
890.00	765.85	762.95	767.36	759.65	757.50	757.48
891.00	765.84	762.89	767.67	759.84	757.46	757.92
892.00	765.48	763.49	768.00	760.04	756.96	757.77
893.00	765.46	763.37	768.00	759.85	757.54	757.38
894.00	765.32	762.82	767.27	759.65	758.24	757.18
895.00	765.51	762.76	767.01	759.23	757.24	756.84
896.00	765.49	762.40	767.26	759.68	756.72	757.41
897.00	765.54	762.44	766.77	759.69	756.76	756.99
898.00	765.61	762.05	767.35	759.71	757.06	757.04
899.00	765.58	762.04	767.45	759.32	756.98	756.87
900.00	765.03	762.04	766.95	758.69	756.68	756.81
901.00	765.16	762.31	767.09	758.70	756.91	756.35
902.00	765.27	762.27	766.76	759.07	756.75	756.99
903.00	765.17	762.07	767.24	758.44	756.61	756.82
904.00	765.02	762.03	766.67	758.76	756.73	756.84
905.00	765.04	761.98	766.99	759.01	756.29	756.86
906.00	765.15	761.39	767.06	758.98	756.64	756.34
907.00	764.38	761.21	766.51	758.78	756.78	756.21

908.00	764.14	761.66	766.59	758.48	756.12	755.85
909.00	764.41	761.46	766.34	758.43	756.01	755.82
910.00	764.60	761.35	765.87	758.22	756.13	756.05
911.00	764.66	761.13	766.32	758.51	755.92	756.28
912.00	764.53	761.49	765.98	758.01	755.92	756.64
913.00	764.03	761.28	766.23	758.21	756.05	755.88
914.00	763.96	760.91	766.08	757.97	756.05	755.60
915.00	763.95	760.97	765.94	758.35	755.41	755.59
916.00	763.89	760.43	765.83	757.75	755.38	755.39
917.00	763.92	760.70	765.85	757.67	755.71	755.68
918.00	763.71	761.03	765.48	757.80	755.51	755.94
919.00	763.38	761.00	765.27	758.24	755.50	755.79
920.00	763.93	760.92	765.36	757.83	755.24	755.35
921.00	764.07	760.63	765.68	757.27	755.30	755.47
922.00	763.43	760.05	765.52	757.26	755.47	755.11
923.00	763.13	760.22	765.34	757.70	754.81	754.99
924.00	763.02	760.26	765.20	757.40	755.04	754.32
925.00	762.79	760.33	764.53	756.93	755.22	754.61
926.00	762.71	760.42	764.72	756.76	755.07	755.03
927.00	763.14	759.90	764.85	756.99	754.96	755.17
928.00	762.86	759.73	764.95	757.11	754.83	755.12
929.00	762.83	759.71	764.65	757.13	754.74	754.59
930.00	762.94	760.01	764.46	756.88	754.83	754.42
931.00	763.39	760.10	764.55	756.70	754.56	754.41
932.00	762.68	759.57	764.62	756.76	754.50	754.46
933.00	762.44	759.74	764.32	756.41	754.06	754.36
934.00	762.43	759.52	764.20	756.51	754.40	754.31
935.00	762.14	759.69	764.09	756.63	754.22	754.42
936.00	762.02	759.18	763.91	756.48	754.27	754.22
937.00	762.10	759.12	763.99	756.59	754.13	753.77
938.00	762.00	759.33	764.25	756.20	754.34	754.28
939.00	762.11	759.55	764.16	755.85	753.80	754.25
940.00	761.54	759.37	763.93	755.83	753.53	753.99
941.00	761.70	759.05	764.05	756.07	753.95	753.78
942.00	761.72	759.05	764.05	755.91	753.98	753.91
943.00	761.85	758.89	763.65	755.77	753.89	754.07
944.00	761.51	758.75	763.47	755.62	753.67	753.68
945.00	761.55	758.88	763.37	755.52	753.73	753.32
946.00	761.48	758.68	763.15	755.91	753.41	752.86
947.00	761.53	758.79	763.74	756.04	753.24	753.21
948.00	761.50	758.63	763.26	755.57	753.09	753.20
949.00	761.26	758.64	763.35	755.17	752.84	753.64
950.00	761.05	758.52	763.64	754.93	752.87	753.36
951.00	761.81	758.31	763.61	754.98	752.91	752.70
952.00	761.60	758.05	763.00	755.14	753.01	752.65
953.00	761.36	758.13	762.56	755.12	752.81	753.06
954.00	760.99	757.72	762.73	754.81	752.57	753.09
955.00	760.64	757.90	762.80	754.76	752.55	752.35
956.00	760.83	757.98	762.95	755.00	752.79	752.70
957.00	761.18	757.71	762.91	754.78	752.45	752.82
958.00	760.58	758.04	762.69	754.80	752.23	752.59
959.00	760.35	758.03	762.33	754.38	752.51	752.57
960.00	760.35	757.74	762.54	754.50	752.73	752.72
961.00	760.52	757.33	762.31	754.75	752.48	752.29

962.00	760.41	757.57	762.19	754.43	751.97	752.23
963.00	760.38	757.41	761.96	754.42	751.66	752.26
964.00	760.46	757.36	762.14	754.02	751.90	751.87
965.00	760.24	757.40	762.07	753.96	752.27	751.95
966.00	759.89	757.00	762.32	754.01	751.88	752.07
967.00	760.13	757.06	761.89	753.90	751.59	752.02
968.00	760.33	757.30	762.10	753.89	751.54	752.10
969.00	760.05	757.21	761.89	753.83	751.71	752.20
970.00	759.65	756.96	762.03	753.72	751.45	751.72
971.00	759.25	756.60	761.61	753.52	751.35	751.49
972.00	759.45	756.75	761.80	753.51	751.47	751.46
973.00	759.42	756.62	761.58	753.72	751.65	751.55
974.00	759.62	756.80	761.26	753.53	751.57	751.55
975.00	759.71	756.47	761.18	753.98	751.37	751.60
976.00	759.54	756.31	761.46	753.43	751.01	751.30
977.00	759.17	756.28	761.25	753.46	750.92	750.95
978.00	758.91	756.36	761.30	753.28	751.14	751.09
979.00	758.81	756.27	761.24	753.12	751.11	751.05
980.00	758.77	755.85	761.00	752.84	751.04	750.95
981.00	759.18	756.10	760.87	752.64	750.64	751.15
982.00	758.87	755.89	760.67	753.18	750.33	750.71
983.00	758.66	755.78	760.76	752.81	750.77	750.32
984.00	758.76	755.73	761.03	752.86	750.89	750.66
985.00	758.82	755.71	760.97	752.86	750.43	750.96
986.00	758.16	755.62	760.74	752.91	750.26	750.43
987.00	757.86	755.44	760.35	752.71	750.19	750.00
988.00	758.11	755.81	760.30	752.30	750.27	750.35
989.00	758.17	755.59	760.20	752.12	750.24	750.01
990.00	758.18	755.69	760.43	752.24	749.77	750.16
991.00	758.24	755.29	760.32	752.33	749.91	750.45
992.00	758.09	755.17	759.96	752.18	749.93	750.16
993.00	758.06	754.96	760.14	751.72	749.92	749.97
994.00	758.30	755.05	759.98	751.74	749.70	749.86
995.00	758.28	755.13	759.70	751.68	749.79	749.53
996.00	757.90	755.00	759.83	752.09	749.87	749.82
997.00	757.54	754.73	760.01	752.19	749.59	749.57
998.00	757.32	754.93	759.80	751.59	749.38	749.66
999.00	757.73	754.78	759.74	751.48	749.20	749.28
1000.00	757.57	755.03	759.74	751.43	749.47	749.09
1000.25	757.51	754.59	759.22	751.28	749.16	749.57
