# Supplementary material for: Atomistic study on mechanical properties of Al matrix composite with different combining forms of reinforcements
Source: PLoS One. 2025 Aug 11;20(8):e0329889. doi: 10.1371/journal.pone.0329889 (PMC12338809; doi:10.1371/journal.pone.0329889)
Supplement: S5 Table — (PDF) [file pone.0329889.s005.pdf]

Strain /(%)	Stress/(GPa)								
	I			II			III		
	x	y	z	x	y	z	x	y	z
0	2353.019	2883.573	2878.096	2206.702	3701.35	1858.482	−357.891	3372.9123	4059.593
0.2	4238.396	4720.979	4706.942	4471.809	5898.387	3763.233	1634.932	5631.3556	6430.283
0.4	6251.324	6828.903	7025.519	6617.741	7982.05	6196.939	4277.302	7865.6671	8369.429
0.6	8450.593	8749.913	9014.754	9005.972	10134.39	8470.434	6752.226	10206.111	10892.29
0.8	10114.63	10602.2	11087.41	11169.43	11987.38	10793.57	8898.97	12377.291	13145.95
1	11796.54	12750.66	12934.23	13199.7	14609.17	12871.48	11214.02	14657.62	14991.5
1.2	13985.58	14284.24	14888.46	15527.96	16347.31	15157.06	13632.33	16445.981	17197.63
1.4	15488.13	15772.11	17108.43	17938.53	18206.9	17255.05	15875.86	18739.783	19478.64
1.6	17365.94	17957.37	19044.03	19799.07	19804.41	18883.08	17776.72	20728.38	21591.95
1.8	19321.63	19505.39	20825.77	21827.47	21672.71	21123.13	19605.48	22729.829	23890.43
2	20809.52	20953.54	22769.8	24002.08	22881.61	22744.95	21484.64	24736.23	25271.31
2.2	22668.51	22948.12	24350.17	25642.82	24324.45	24331.84	23230.11	26257.362	27354.81
2.4	24205.51	24463.28	26085.2	27428.13	26114.49	25634.88	25229.63	27490.033	29074.39
2.6	25991.45	26208.13	27915.1	29188.58	28327.12	27858.27	26995.45	28768.017	30073
2.8	27390.23	27651.2	29522.9	30855.67	29555.06	29457.58	28330.77	29968.565	31242.33
3	28936.69	29336.65	31004.75	32223.1	31349.66	31306.27	30694.99	30407.909	32059.19
3.2	30522.12	30669.92	32803.28	33186.36	32913.93	32817.33	32258.27	31452.656	33691.99
3.4	32028.87	31860.96	34111.93	34532.04	34280.43	34596.66	33840.96	32912.046	34789.58
3.6	33394.75	33586.86	35725.46	36096.46	34885.55	36204.68	35311.52	34780.495	36079.69
3.8	35203.08	35038.68	36914.59	37185.82	35903.58	37998.43	36962.62	36139.65	37665.13
4	36453.02	36366.8	38722.49	38778.66	37415.91	39609.7	38041.73	37760.244	39297.8
4.2	37528.72	37815.45	40078.91	40011.83	38764.31	41212.24	39500.98	39155.991	40835.19
4.4	38869.69	38940.11	41071.51	41458.76	39625.03	42446.59	40783.06	40851.527	42579.65
4.6	40155.83	40269.12	42470.87	42862.67	41238.5	44077.16	42177.68	41961.065	44028.61
4.8	41227.83	41363.95	43484.41	44486.78	42283.19	45347.59	43356.13	43196.796	45932.47
5	42652.72	42757.21	44727.39	46069.09	43532.58	47085.56	44897.34	44849.952	47202.43
5.2	44013.37	43839.74	46340.36	47534.44	44876.75	48646.11	45947.38	46167.923	48433.9
5.4	45056.16	45102.65	47288.48	48844.76	46363.95	49801.18	47513.56	47418.383	49555.61
5.6	46477.3	46025.33	48402.26	50157.42	47581.56	51176.37	49059.32	48883.547	50813.28
5.8	47358.33	47010.13	49740.27	51609.22	48629.12	52406.11	50553.24	50427.492	51463.43
6	48728.73	47838.81	50525.68	52710.16	49616.48	53510.02	51704.87	51357.917	51462.38
6.2	49748.94	49018.65	51453.34	54043.7	50637.4	54490.87	53109.46	52314.847	51666.51
6.4	50860.27	49963.78	52555.47	54839.38	51698.34	55223.71	54233.75	52944.918	51565.97
6.6	51704.07	51385.94	53378.21	55418.88	53468.75	56288.13	55073.32	53901.994	52503.01
6.8	52660.42	52222.53	54370.36	56649.89	54698.2	57041.25	56174.8	54715.897	53373.47
7	53546.17	53214.78	55494.16	57552.87	55786.94	58601.28	56719.52	55226.621	54157.22
7.2	54978.56	54328.13	56323.67	58177.07	57342.5	59857.6	57848.59	56066.891	55350.5
7.4	55549.29	55310.17	57229.97	58448.11	58614.05	60726.67	58291.18	57204.5	56509.52
7.6	56317.93	55948.73	58088.56	59503.34	59822.12	61772.99	59279.81	58494.541	57838.77
7.8	57378.07	56889.42	58973.9	59985.51	60997.11	62640.34	60521.83	59419.328	59361.25
8	58288.74	57870.58	59832.06	60895.84	62119.72	63718.12	61658.53	60490.92	60410.5
8.2	59047.3	58881.3	60659.74	62014.62	62679.45	64754.91	62809.38	61486.442	61572.82
8.4	59985.34	59734.39	61878.92	62953.97	63739.61	65583.41	63964.03	62522.757	62883.56
8.6	60876.49	60461.54	62754.48	63692.54	64468.59	66254.53	65082.9	63799.234	63891.75
8.8	61336.91	61357.63	63186.69	64842.13	65300.49	67244.64	66306.72	64776.482	64814.75
9	62178.37	62176.67	64261.52	65728.92	66077.32	67991.45	66881.01	65536.731	65582.86
9.2	63511.03	62978.85	64737.54	66625.39	66825.23	68510.87	66946.55	66484.522	66303.08
9.4	64088.24	63771.42	65368.03	67715.78	67599.59	69097.47	67371.41	66958.223	66377.47
9.6	64923.4	64482.86	66389.66	68532.67	68147.99	69180.98	67709.33	67444.227	66652.58

9.8	65840.35	65589.81	66910.07	69327.59	68242.75	68584.28	68374.14	68088.97	66672.51
10	66636	66220.55	67493.21	69573.41	68329.95	66894.4	69231.32	68694.373	67304.75
10.2	67269.1	67188.07	68301.68	69473.72	67421.32	62428.33	70211.18	68690.486	67648.7
10.4	68098.12	68193.42	68777.22	68972.99	66909.88	56217.54	70450.99	68026.033	68262.24
10.6	68891.72	68606.2	69184.39	66606.5	66385.05	46298.61	70075.76	66560.846	68869.98
10.8	69545.94	69400.95	69471.68	61718.54	64926.18	36660.07	68771.13	63815.647	68613.03
11	69809.69	70214.98	70243.89	54560.01	62410.6	30838.69	65521.34	59332.45	68091.62
11.2	68506.28	70747.87	70927.05	46017.8	58610.36	28451.24	59041.88	53627.147	65158.38
11.4	63023.78	70420.14	71554.56	38157.63	52682.36	26910.96	50733.61	45566.514	60232.62
11.6	51236.35	69249.01	71873.95	33431.05	45375.98	25604.24	42246.18	37624.812	52940.12
11.8	35949.19	63711.92	69843.45	30733.64	38504.19	26187.82	36709.95	32100.706	45536.58
12	26814.83	52245.3	62343.65	28833.49	32743.88	25979.14	33148.66	30361.853	38264.62
12.2	25421.54	38810.28	48458.24	27872.1	28592.9	24133.33	30830.77	29153.96	33748.74
12.4	24945.53	30618.82	34250.42	26616.5	26319.53	23896.6	29103.13	28888.093	29808.19
12.6	24860.01	26870.86	28196.19	25344.6	24873.55	24070.65	28281.31	27633.301	28202.46
12.8	23844.34	25581.18	26456.07	24054.33	24144.62	24229.07	27265.28	27588.768	26999.24
13	24063.86	25135.78	24225.3	23735.51	23382.81	23674.68	26176.8	26649.832	26256.59
13.2	23506.88	23977.96	23101.86	23907.98	23674.64	24267.28	25626.67	26564.798	26109.38
13.4	23509.66	23870.96	22571.77	24153.88	23411.51	23848.5	25584.59	25450.749	25853.94
13.6	23904.63	23723.24	22360.83	24768.3	23736.49	23852.12	25063.96	26181.896	25021
13.8	23227.27	23059.69	22614.58	24284.44	23605.7	23581.63	24828.11	25726.463	25677.41
14	23121.16	23594.54	22723.8	23612.2	23731.03	23966.16	24638.45	25446.485	24990.9
14.2	23449.19	23073.54	22154.04	24072.64	23507.77	23390.3	23858.46	25385.523	24470.65
14.4	23565.96	22998.17	22492	24512.58	23803.37	23317.35	23511.5	24845.696	24886.58
14.6	23418.6	21981.55	22508.61	24351.92	23773.99	23272.29	25028.05	25511.245	24871.74
14.8	23990.52	22362.46	22957.61	24146.5	23645.8	23280.19	23767.93	25954.112	24775.97
15	24157.55	22213.7	23175.82	25399.72	23830.89	24317.81	24012.16	26174.43	24178.53
15.2	24555.46	22112.05	23109.34	24663.28	24461.27	23546.96	23269.87	26080.589	24219.87
15.4	24521.59	21576.17	22923.6	24286.96	24401.51	23669.59	22828.42	24658.701	24010.15
15.6	24841.24	21617.12	22921.1	24518.4	25283.55	23458.84	22708.85	25127.66	23663.21
15.8	24770.7	21824.91	22727.57	23904.93	24707.32	23108.08	22581.93	24848.164	23901.24
16	24364.35	22168.66	22156.23	24378.41	24799.16	22681.52	22772.59	25216.044	23426.94
16.2	24132.13	21815.95	22928.76	23932.51	23977.23	22958.13	22323.36	24603.193	23260.71
16.4	23303.79	21648.59	22562.32	23770.03	23827.97	22536.65	21358.63	24411.239	23248.97
16.6	23847.63	21405.34	22639.25	23160.97	23832.91	22279.83	21526.83	23787.859	23273.95
16.8	23983.76	21071.96	23064.28	22919.27	23104.3	22830.65	21254.75	23647.403	23128.94
17	23815.95	21178.48	22994.38	23001.71	22414.06	22434.42	21977.65	22737.949	22550.07
17.2	23429.53	22017.41	23652.22	22789.14	22440.59	22458.83	21925.17	23070.578	22394.31
17.4	23002.87	21017.17	23803.52	23211.86	21644.38	22688.09	21867.27	22657.396	21716.24
17.6	22416.61	20807.13	23254.61	22800.07	22509.01	22279.98	21837.26	22075.229	21194.97
17.8	22938.85	20541.68	23267.94	22915.8	21829.46	21830.85	21166.86	23157.109	20576.66
18	22982.23	20311.55	22917.16	22238.53	21234.71	21626.78	20363.07	22572.025	20419.41
18.2	23068.67	20404.41	22514	21851.17	20329.15	21190.53	21302.07	22412.39	20285.58
18.4	22944.93	20290.73	22680.86	22184.66	20221.23	20749.11	20137.04	22672.912	20779.07
18.6	22771.57	19921.56	22016.45	21700.17	20185.21	20939.43	20144.69	22267.356	20243.03
18.8	22219.02	19744.75	21928.26	21397.81	19913.02	21186.95	19960.94	22295.656	20096.87
19	22684.68	19762.97	21286.55	21304.68	19294.09	20217.16	19815.8	22926.285	20208.99
19.2	22559.13	19532.72	21658.15	21398.93	20223.15	21375.17	19730.57	22370.397	20230.94
19.4	22400.63	19542.88	21315.69	20192.45	20205.35	20907.74	19629.69	21183.903	20021.89
19.6	22570.18	19475.53	21144.08	19983.79	20169.83	21206.8	19964.5	21304.344	20097.19
19.8	22149.79	19806.59	21006.67	19396.55	20937.36	21502.15	19822.13	20983.465	19641.62
20	22085.86	20035.69	20222.61	19504.27	20995.79	21563.47	20023.58	20573.823	19952.35

Strain /(%)	Stress/(GPa)								
	Ⅳ			Ⅴ			Ⅵ		
	x	y	z	x	y	z	x	y	z
0	2724.621	2905.714	1636.352	3003.41	2930.484	1396.305	3118.106	2875.9	1802.994
0.2	4734.089	4720.392	4006.738	5016.077	5006.595	3108.441	4991.714	5130.881	3766.336
0.4	7037.742	6927.989	6035.321	7377.216	7132.081	5682.273	7209.663	7165.537	6019.005
0.6	8805.469	9280.065	8139.737	9639.963	9243.278	7912.294	9316.954	8980.009	8061.641
0.8	10965.58	10929.97	10241.11	11926.11	11234.76	10042.51	11410.66	11049.01	9980.241
1	12711.57	13148.14	12431.32	13935.26	13395.47	12207.74	13415.73	13189.92	12284.63
1.2	14516.5	14917.06	14354.55	15928.81	15310.17	14400.58	14952.66	15270.59	14096.98
1.4	16806.65	16727.9	16456.62	18182.62	17717.95	16353.06	16701.15	17208.94	15880.02
1.6	18708.88	18688.87	18614.22	19879.87	19517.36	18260.92	18902.06	18775.79	17995.45
1.8	20278.7	20135.83	20407.47	21690.66	21264.77	20372.79	20397.85	20494.1	19320.02
2	22248.45	22079.82	22201.76	23339.76	22955.03	22125.91	22129.3	22415.58	21118.06
2.2	23637.71	23828.55	23643.41	25368.24	24426.91	24024.35	23879.33	23947.56	23141.32
2.4	25343.67	25824.2	25442.29	27426.92	26536.77	26002.7	25744.35	25373.53	24870.33
2.6	27458.81	27168.43	27221.5	29084.93	28279.93	27719.58	27059.16	27298.32	26329.13
2.8	28858.05	28720.37	28923.33	30164.42	30188.38	29302.86	28608.44	28609.76	27516.18
3	30334.7	30658.96	30271.62	31367.17	31572.37	30865.62	30410.65	30151.14	28902.13
3.2	31944.72	32254.43	31841.02	32881.97	33031.22	32580.71	31599.08	31467.92	30039.37
3.4	33376.94	33737.32	33350.66	34092.59	34661.72	34299.55	32957.93	32879.3	31487.84
3.6	34835.08	35247.76	34978.26	35390.64	36107.56	35871.63	33989.16	34110.85	33350.31
3.8	36543.57	36675.9	36289.51	36745.16	37388.01	37568.52	34945.94	35240.79	34616.3
4	37668.03	37773.17	37790.66	37711.26	38527.59	39156.35	36523.45	37070.62	36096.87
4.2	39387.79	38674.03	39583.91	38646.71	39457.07	40249.26	38281	38239.62	37512.17
4.4	40314.11	40127.47	40695.71	40289.51	40666.02	41136.01	39655.99	39805.93	38897.07
4.6	41869.68	41532.39	42170.7	41816.08	41089.12	43268.06	40663.3	41186.6	40013.94
4.8	43469.94	42776.92	43394.85	43212.66	41711.95	44279.18	42056.81	42756.49	41586.32
5	44383.93	43873.85	44692.14	44702.41	43132.16	44792.88	43671.79	43752.36	42974.13
5.2	45533.06	45183.7	45521.33	45959.04	44358.04	45669.9	44972.02	45090.02	43854.6
5.4	47005.38	46229.96	46392.55	47598.26	45772.07	46948.54	46160.86	46189.93	45223.21
5.6	48427.81	47332.29	47670.28	49060.44	46729.65	48565.26	47578.72	47290.93	46539.57
5.8	49350.87	48720.18	48905.63	50345.07	48220.95	49946.89	49040.34	48037.52	47591
6	50521.09	50081.12	49870.13	51924.94	48771.46	51085.76	50115.33	49061.43	48858.2
6.2	51711.89	50850.59	51142.7	53216.77	50103.58	52059.34	51542.99	50235.37	50015.67
6.4	52776.86	51936.82	51930.29	54160.89	51364.23	52542.34	52707.95	51027.41	51484.46
6.6	54051.85	53122.71	53241.64	55301.88	52358.93	53921.68	53012.18	51906.8	52346.54
6.8	54479.47	54037.22	54009.9	55904.74	53433.18	54926.15	54031.39	53097.54	53584.48
7	55648.09	54842.97	54323.15	57232.71	54157.53	56181.91	55272.97	54216.59	54493.24
7.2	56727.93	55851.64	55066.83	58002.57	55221.93	56893.69	56277.29	54934.32	55316.25
7.4	57275.79	56735.74	55436.67	58925.49	56053.83	57410.16	57184.85	56059.36	56038.34
7.6	58141.87	57649.84	56368.27	59429.06	56273.21	58138.02	57808.22	56988.7	57135.83
7.8	59084.53	58115.94	57345.43	60457.25	57136.9	59116.04	58495.45	58044.43	57618.21
8	59818.79	58726.06	58117.9	60786.88	58124.66	59919.59	59527.98	59016.42	58627.31
8.2	61124.6	59768.24	58945.99	61683.48	59210.45	60779.22	60332.39	59937.04	59816.2
8.4	61392.36	60938.36	59961.52	62061.73	60301.37	61885.53	60920.3	60779.89	60397.98
8.6	62178.4	61437.4	61360.08	62158.99	60837.08	62515.81	61598.28	61764.13	61207.38
8.8	62960.07	62557.07	62306.7	62909.52	61597.83	63146.15	62266.74	63226.87	62253.26
9	63830.04	63658.56	63261.32	63825.1	62626.9	64545.69	63609.14	64155.66	63101.46
9.2	64848.82	64221.4	64139.38	64343.37	63556.22	65269.52	64759.23	65115.13	63477.89
9.4	65487.04	65030.26	65039.79	65601.51	64589.03	66256.31	65547.89	66006.8	64000.48
9.6	66238.61	65813.18	65984.6	66601.94	65409.43	66915.28	66207.38	66478.23	64329.21

9.8	66269.49	66161.56	67016.39	67223.63	66548.23	66437.35	66986.8	66955.92	64082.01
10	66375.36	67185.83	67362.49	68375.3	67657.04	65264.32	67596.38	67470.64	62692
10.2	66324.13	67658.23	67558.28	69029.99	68227.52	62030.62	68723.75	67853.64	59374.2
10.4	64884.67	67204.83	66827.58	69137.87	69075.81	56971.83	69431.37	68181.21	53705.36
10.6	60930.14	64283.28	64530.42	69623.58	70184.81	49873.77	70063.51	68579.5	46509.77
10.8	53352.66	57026.53	59491.13	70396.81	70735.67	41809.27	70594.9	68276.25	40716.48
11	43856.27	44898.52	51195.53	70762.95	70153.19	37140.82	71035.03	66532.99	35549.33
11.2	35019.5	35645.58	40763.29	69523.08	68691.77	33061.21	69884.64	62360.94	32337.99
11.4	29025.11	29136.39	32136.12	67615.85	64581.68	29695.92	66922.76	54152.09	30319.49
11.6	26255.31	27006.26	27376.62	64407.53	57715.35	28390.79	61684.11	42023.1	28711.92
11.8	23898.32	25421.79	24892.89	60074.63	48624.83	27448.51	53722.69	32150.22	26737.33
12	23705.87	24703.74	23091.21	53870.64	39842.04	26788.07	43630.67	27704.41	25644.68
12.2	22920.62	24104.64	23015.89	46883.04	34391.28	26273.61	34643.64	25243.56	25039.63
12.4	22352.84	24104.76	23200.79	40816.76	31093	25301.56	28981.31	25436.92	24224.57
12.6	22823.25	23976.34	22524.72	35732.87	28561.41	24982.43	26498.22	24494.6	24219.04
12.8	22172.69	23964.73	22855.58	30798.16	28216.74	25265.26	25759.38	24083.79	24448.14
13	22316.75	23509.74	22425.45	29129.51	27232.8	25307.37	26117.89	23960.4	24748.37
13.2	23240.81	23298.53	22618.85	28246.45	26381.22	25323.92	25687.47	24169.59	24142.09
13.4	22603.01	23772.96	22752.47	26773.46	25647.05	24980.72	24984.82	23772.44	24243.79
13.6	22355.62	23338.5	23165.05	26264.39	25272.16	24671.12	24779.06	23821.58	24107.3
13.8	22121.36	22631.54	23399.2	25927.25	24500.57	24831.83	25438.71	22814.01	24091.31
14	22627.36	22981.67	23294.52	25913.17	24230.72	24328.2	25148.34	22974.76	24630.14
14.2	23166.49	22753.67	23236.19	25112.87	24150.17	24314.71	25757.23	22876.65	24959
14.4	23401.25	22371.68	24182.36	24992.1	24259.02	22851.87	24809.3	22901.54	25403.37
14.6	23656.46	22648.49	23532.35	24486.17	24087.77	22593.03	25424.99	22755.08	25139.98
14.8	23525.74	22360.43	23910.71	23876.62	24530.88	22552.3	25300.31	22952.45	25582.89
15	23321.69	21914	24449.53	24681.2	24173.15	22454.8	25661.55	22307.48	25212.88
15.2	23117.9	21903.2	24321.41	24657.09	23675.51	21919.7	25362.43	22263.21	25602.01
15.4	23744.08	21513.71	23781.69	23769.24	23157.94	22254.98	25613.81	21710.21	24401.86
15.6	23096.91	21910.83	24366.66	23354.87	23216.53	22313.1	26371.62	21785.34	23796.01
15.8	23817.88	21514.6	23972.31	22930.46	22871.2	22109.1	26160.2	21726.86	22917.06
16	23365.27	21643.63	23438.7	21698.31	23424.6	21467.99	26022.14	21329.97	22770.81
16.2	23394.74	21115.28	23067.51	21911	23535.34	22043.18	25653.16	21260.33	21659.4
16.4	23782.26	20344.76	22738.55	21594.95	23283.64	21602.35	25976.99	21200.57	21830.74
16.6	23497.43	19953.41	23071.3	21818.78	23538.06	21713.2	25883.46	20495.28	20552.19
16.8	22914.49	19766.41	22860.4	22502.33	23931.02	21476.44	26450	20951.35	20206.82
17	22150.62	19811.41	21698.67	21486.61	23858.81	20247.3	26885.18	21500.34	20544.44
17.2	21927.95	19989.72	21235.94	22499.01	24213.35	19632.5	26538.1	20348.73	20805.96
17.4	20824.67	18903.16	21273.7	22251.81	23939.86	18634.77	26167.27	20301.46	21076.83
17.6	20079.68	19565.99	20776.42	22385.83	23569.33	18039.45	25668.8	20718.65	20473.53
17.8	20068.56	18612.35	20600.47	22130.3	24075.95	18609.19	25383.98	20459.98	20067.72
18	20262.43	18462.16	19697.89	21988.05	23576.98	18974.19	24401.03	19695.77	19487.45
18.2	20033.27	18388.03	20123.39	22057.58	23721.55	19317.48	23917.12	19501.25	19285.18
18.4	19990.05	17471.97	20133.7	21873.3	22968.32	18340.64	23752.78	19240.53	19657.38
18.6	20345.6	17199.43	19791.27	22544.05	22415.2	18736.63	23182.32	18575.76	19958.3
18.8	20266.57	17707.86	19872.5	21770.21	22089.98	18794.56	23059.93	18563.73	19922.22
19	20522.85	17161.47	19364.79	22776.88	20701.07	18844.51	22850.14	19076.02	19591.17
19.2	20399.55	17462.23	19912.89	22768.66	20545.02	18376.36	23244.34	18976.59	19438.27
19.4	20380.03	17715.86	19725.61	23112.87	20549.61	18165.11	23234.96	18683.69	19056.06
19.6	19430.32	16762.09	20143.72	22838.04	19956.81	17975.56	23617.17	19021.27	18366.48
19.8	19047.25	17075.97	19979.27	23070.92	20257.3	18079.89	23285.73	18632.76	17671.12
20	18608.74	16423.85	19680.91	22261.8	20014.17	18229.88	22952.58	18198.3	17266.52
