# Supplementary material for: Atomistic study on mechanical properties of Al matrix composite with different combining forms of reinforcements
Source: PLoS One. 2025 Aug 11;20(8):e0329889. doi: 10.1371/journal.pone.0329889 (PMC12338809; doi:10.1371/journal.pone.0329889)
Supplement: S6 Table — (DOCX) [file pone.0329889.s006.docx]

Table 1. Structural analysis and mechanical properties of sintered models.

|  | Al Volume  /(nm^3^) | Pore volume  /(nm^3^) | The ratio of arranged Al atoms  /(%) | The total tensile strength  /(GPa) | The total elongation at maximum stress  /(%) |
| --- | --- | --- | --- | --- | --- |
| I | 746.96652 | 0.11138 | 64.73493 | 21.24315 | 33.8 |
| II | 744.04313 | 0.07763 | 84.55691 | 20.70843 | 29.6 |
| III | 744.09012 | 0.06075 | 89.41875 | 20.80153 | 31 |
| IV | 745.33529 | 0.06413 | 64.50754 | 20.15919 | 30.4 |
| V | 743.17427 | 0.14175 | 84.61972 | 20.84139 | 31.4 |
| VI | 743.59904 | 0.15862 | 84.99653 | 20.39437 | 31.2 |

Table 2. Mechanical properties of sintered models along different axis.

|  | The tensile strength along different axis  /(GPa) | | | The total elongation at maximum stress along different axis  /(%) | | |
| --- | --- | --- | --- | --- | --- | --- |
|  | X | Y | Z | X | Y | Z |
| I | 6.98097 | 7.07479 | 7.18739 | 11 | 11.2 | 11.6 |
| II | 6.95734 | 6.83299 | 6.9181 | 10 | 10 | 9.6 |
| III | 7.0451 | 6.86944 | 6.887 | 10.4 | 10 | 10.6 |
| IV | 6.63754 | 6.76582 | 6.75583 | 10 | 10.2 | 10.2 |
| V | 7.07629 | 7.07357 | 6.69153 | 11 | 10.8 | 9.6 |
| VI | 7.1035 | 6.85795 | 6.43292 | 11 | 10.6 | 9.6 |

Table 3. Thermal properties of sintered models along different axis.

|  | The thermal conductivities along different axis  /(W/mK) | | |
| --- | --- | --- | --- |
|  | X | Y | Z |
| I | 237.09318 | 217.22949 | 221.17191 |
| II | 254.76854 | 251.29501 | 306.92375 |
| III | 308.33627 | 311.96712 | 307.58961 |
| IV | 261.77114 | 232.96034 | 253.9231 |
| V | 279.83287 | 270.12098 | 288.18487 |
| VI | 301.96223 | 274.8977 | 307.1787 |
